# Dpp Signaling Activity Requires Pentagone to Scale with Tissue Size in the Growing *Drosophila* Wing Imaginal Disc

**DOI:** 10.1371/journal.pbio.1001182

**Published:** 2011-10-25

**Authors:** Fisun Hamaratoglu, Aitana Morton de Lachapelle, George Pyrowolakis, Sven Bergmann, Markus Affolter

**Affiliations:** 1 Growth & Development, Biozentrum, University of Basel, Basel, Switzerland; 2 Department of Medical Genetics, University of Lausanne, Lausanne, Switzerland; 3 Swiss Institute of Bioinformatics, Lausanne, Switzerland; 4 Institute for Biology I, Albert-Ludwigs-University of Freiburg, Freiburg, Germany; 5 Centre for Biological Signaling Studies (BIOSS), Albert-Ludwigs-University of Freiburg, Freiburg, Germany; Stanford University, United States of America

## Abstract

The wing of the fruit fly, *Drosophila melanogaster*, with its simple, two-dimensional structure, is a model organ well suited for a systems biology approach. The wing arises from an epithelial sac referred to as the wing imaginal disc, which undergoes a phase of massive growth and concomitant patterning during larval stages. The Decapentaplegic (Dpp) morphogen plays a central role in wing formation with its ability to co-coordinately regulate patterning and growth. Here, we asked whether the Dpp signaling activity scales, i.e. expands proportionally, with the growing wing imaginal disc. Using new methods for spatial and temporal quantification of Dpp activity and its scaling properties, we found that the Dpp response scales with the size of the growing tissue. Notably, scaling is not perfect at all positions in the field and the scaling of target gene domains is ensured specifically where they define vein positions. We also found that the target gene domains are not defined at constant concentration thresholds of the downstream Dpp activity gradients P-Mad and Brinker. Most interestingly, Pentagone, an important secreted feedback regulator of the pathway, plays a central role in scaling and acts as an expander of the Dpp gradient during disc growth.

## Abstract 2

The activity of the Dpp morphogen adapts to tissue size in the growing *Drosophila* wing imaginal disc, and Pentagone, an important secreted feedback regulator of the Dpp pathway, is required for this adaptation.

## Author Summary

Scaling, the fitting of pattern to size, manifests itself in numerous examples around us. During development, individual body parts scale up to fit the overall body size. Starved animals form smaller adults with proportionally smaller parts, and amphibian embryos can form normally proportioned adults after extreme surgical operations. How scaling is achieved is not well understood. Here, we establish the *Drosophila* wing imaginal disc, the precursor tissue of the adult wing, as a model to study scaling quantitatively during growth. In this model, we define scaling as the preservation of proportions of gene expression domains with tissue size during disc growth. The Decapentaplegic (Dpp) morphogen is known to play a central role in Drosophila wing formation and co-coordinately regulates growth and patterning. We found that as the disc grows, the Dpp response expands and scales with the tissue size. Interestingly, scaling is not perfect at all positions in the field. The scaling of the target gene domains is best where they have a function; Spalt, for example, scales best at the position in the anterior compartment where it helps to form one of the anterior veins of the wing. Analysis of mutants for *pentagone*, a transcriptional target of Dpp that encodes a secreted feedback regulator of the pathway, indicates that Pentagone plays a key role in scaling the Dpp gradient activity.

## Introduction

Matching of pattern to size, a phenomenon referred to as scaling, manifests itself in numerous examples around us. During development, individual body parts scale up with the overall body size, starved animals form smaller adults with proportionally smaller body parts [1],[2], and amphibian embryos can form normally proportioned adults after extreme surgical operations [3]. Also, retardation of growth in *Drosophila* wing imaginal discs, the larval precursors of the adult wings, slows down the growth of the rest of the body [4]. Similarly, experimental reduction of growth rates in part of the wing disc leads to proportional growth defects in the rest of the tissue, and the final organ, though smaller, conserves its proportions [5]. How scaling is achieved is an intriguing question that has long fascinated biologists [6],[7],[8],[9]. Recent findings started shedding light onto this question [2],[4],[5]. Here, we define scaling as the preservation of proportions across different sizes during organ growth, identify an important factor in this process, and establish the *Drosophila* wing imaginal disc as a model to study scaling quantitatively and at the molecular level.

The fruit fly *Drosophila melanogaster* represents an excellent model system for quantitative analyses as it can be manipulated at will using its exquisite genetic tool kit. The positions of the veins in the adult wing scale rather precisely with the final wing size, presumably ensuring that the wing is functional [10],[11]. This observation indicates that there are active mechanisms that coordinate growth and patterning of the wing. The easiest imaginable way of coordinating growth and patterning is by having the same molecules control both processes. *Drosophila* Decapentaplegic (Dpp), a TGF-β superfamily member, is essential for the formation of all imaginal discs [12]. Dpp signaling has been extensively studied in the wing imaginal disc. In this tissue, Dpp is produced in a stripe of cells anterior to and abutting the anterior/posterior (A/P) compartment boundary, spreads into both compartments to form a gradient, and patterns the growing tissue (Figure S1). Dpp is a morphogen with the capability to specify distinct target gene expression domains at different distances from its source. The boundaries of these domains are instrumental in setting the positions of veins during subsequent development of the wing imaginal disc (Figure S1C) [13],[14],[15]. This patterning function of Dpp coupled to its ability to promote growth [16],[17],[18] make Dpp an attractive candidate for being involved in scaling.

The Dpp signal transduction pathway is highly conserved and relatively simple (Figure S1B). Ligand-mediated receptor activation induces phosphorylation of Mothers-against-Dpp (Mad, P-Mad in its phosphorylated and active form) and nuclear translocation of heteromeric complexes of P-Mad and the co-Smad Medea. These complexes directly regulate the expression of a large number of target genes and have the ability to activate as well as suppress transcription [14]. One of the most critical functions of Dpp signaling is to suppress *brinker* (*brk*) transcription because Brk acts as a potent transcriptional repressor of many Dpp target genes (Figure S1) [19],[20],[21]. Repression of *brk* is achieved via short “silencer elements” (SEs) in the *brk* enhancer; the *Drosophila* Smad proteins P-Mad and Medea bind as a trimer (two Mad, one Medea) to the SEs and recruit the co-repressor Schnurri (Shn) [22],[23],[24]. Consequently, the extracellular Dpp gradient and the resulting intracellular P-Mad gradient are translated into an inverse nuclear gradient of Brk [25]. In the lateral regions of the wing disc, where Dpp signaling is relatively low, the Brk gradient delimits the expression domains of the Dpp target genes *daughters-against-dpp* (*dad*), *spalt* (*sal*), and *optomotor blind* (*omb*) (Figure S1). In patches of marked cells where *brk* function is deleted (i.e. *brk* loss of function clone), *dad*, *sal*, and *omb* are derepressed [14],[19],[21]. The P-Mad/Medea complex can also directly bind to and activate transcription of *dad* and *sal* [26],[27]. Hence *dad* and *sal* enhancers read both P-Mad and Brk levels, and their sensitivity to these two factors as well as others appears to determine their expression domains.

While the role of Dad is less well studied, Sal and Omb expression boundaries are crucial for the determination of the positioning of veins L2 and L5 of the adult wing, respectively (Figure S1C) [28],[29],[30]. How are the positions of these veins determined? The pouch section of the wing imaginal disc, which will give rise to the adult wing, is subdivided into alternating vein and intervein territories during the larval stages (Figure S1C). The combined activity of the Epidermal Growth Factor, Notch, Hedgehog, and Dpp pathways culminate in the restricted expression patterns of transcription factors that identify different veins. For example, the zinc-finger proteins Knirps and Abrupt are expressed and required specifically in L2 and L5, respectively. Loss of function mutations of these genes result in the loss of the corresponding veins [28],[31]. Knirps is expressed within the anterior edge of the Sal expression domain, while the L5 primordium forms within the posterior edge of the Omb domain adjacent to cells expressing high levels of Brk (Figure S1C) [28],[29]. Hence, Sal, Omb, and Brk play instrumental roles in setting the positions of L2 and L5 under the control of the Dpp activity gradient.

Recently, *pentagone* (*pent*) emerged as an important target gene of Dpp signaling, playing essential roles for both growth and patterning functions of the pathway. Pent is secreted and directly interacts with the heparan sulfate proteoglycan Dally to promote long-range distribution of the Dpp ligand. Absence of *pent* causes a severe contraction of the Dpp activity gradient resulting in growth and patterning defects of the adult organ. *pent* transcription, like *brk*, is directly repressed by Dpp signaling via SEs and acts as an inbuilt feedback loop with a crucial role in shaping and fine-tuning the Dpp morphogen gradient readout (Figure S1) [32],[33].

Here, we made use of this wealth of information available with regard to the molecular readout of the Dpp signaling activity in the wing imaginal disc and investigated whether the Dpp activity gradients, namely P-Mad and Brk, as well as the downstream domain boundaries (Dad, Sal, and Omb) scale and thus adapt to the size of the growing tissue. After establishing a protocol to reliably quantify the spatial and temporal changes in the Dpp activity gradients, we found that both P-Mad and Brk scale rather well with the tissue size.

We then tried to uncover the molecular mechanisms that ensure proper scaling of these activity gradients. A recent mathematical model termed *expansion-repression integral feedback control* suggested that scaling emerges as a natural consequence of a feedback loop which is based on two diffusible components: a morphogen and a hypothetical molecule termed *expander* [34]. The expander facilitates the spread of the morphogen and in turn is repressed by it, and therefore only produced far away from the morphogen source. As a consequence, the gradient expands as long as the expander molecule is produced. The gradient stops expanding once the morphogen levels are high enough to completely inhibit expander production in the whole field. Because the expander molecule is assumed to be stable and diffusible, the morphogen gradient remains expanded, even when no more expander is produced. In the context of a slowly growing tissue, more expander could be produced in the lateral regions as the tissue grows. The morphogen gradient would thus expand, until expander production would again be inhibited in the entire field. Since Dpp signaling negatively controls the expression of Pent, which itself positively regulates the Dpp activity gradient, we tested whether Pent might act as an expander of the Dpp gradient during disc growth. Our results suggest that Pent indeed plays a role in scaling the Dpp activity gradient.

**Figure 1:**
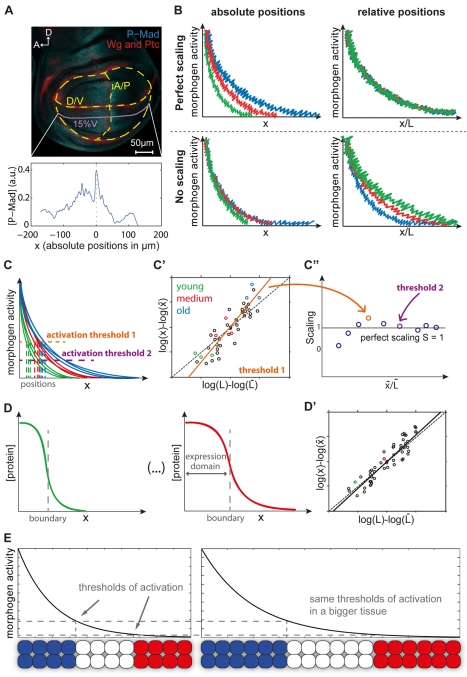
Assessing scaling. (A) Dashed lines outline the pouch, A/P, and D/V boundaries, defined by Wg and Ptc stainings (red). P-Mad (blue) profiles were extracted with 15% ventral offset from D/V (purple). (B) *Qualitative* scaling of activity gradients (P-Mad and Brk). In case of perfect scaling, the profiles expand as the discs grow (top left), and overlap in relative positions x/L (top right). In the absence of scaling, the profiles do not expand as the discs grow (bottom left); thus, when plotted in relative positions (bottom right), they are pulled apart. (C–C″) *Quantitative* scaling of activity gradients. (C) For a given activation threshold, the corresponding positions are retrieved from each profile. (C′) For that threshold, we plot the positions *x* against the disc sizes *L*. Linear regression yields a scaling coefficient for the average relative position 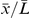 (solid line; the dashed diagonal corresponds to perfect scaling, *S* = 1). Colored circles refer to the profiles in C. (C″) We repeat this procedure for several activation thresholds and obtain a position-dependent picture of scaling. Black line indicates perfect scaling. Arrows indicate the scaling coefficient obtained from (C′) (orange), and for another threshold (purple). (D–D′) *Quantitative* scaling of gene expression domains. (D) For each disc, we determine the boundary position *x* of a domain and the disc size *L*. (D′) We plot the boundary positions as a function of the disc sizes and determine scaling by linear regression. Colored circles refer to the profiles in D. (E) A morphogen or activity gradient is read out in the target field, establishing a pattern. In a bigger tissue where the gradient scales, the resulting pattern keeps the same proportions (1/3 of blue, red and white) and thus scales perfectly, provided that the boundaries are defined at the same threshold concentrations. This model is known as the French flag [35],[36].

The Dpp activity gradient is read out by several target genes, such as *dad*, *sal*, and *omb*, domains of which, we found, scale with tissue size. How is scaling transmitted from the activity gradients to the target gene domains? Inspired by the French flag model for pattern formation [35],[36], we tested whether the target genes *dad*, *sal*, and *omb* respond to similar concentration thresholds of P-Mad and Brk activities during disc growth. In this case, provided that the activity gradients scale, the boundaries characterized by these constant thresholds would shift as the gradient expands, ensuring perfect scaling of the target gene domains with tissue size (Figure 1E). Interestingly, our results do not support such a model, but rather suggest that P-Mad and Brk activity gradients are combined in a gene-specific fashion to ensure proper scaling of the targets. Finally, we compared our dataset to a similar dataset that was recently used to propose a uniform growth model in the wing imaginal disc [37].

## Results

### Generating a Dataset for Quantitative Analysis

**Figure 2:**
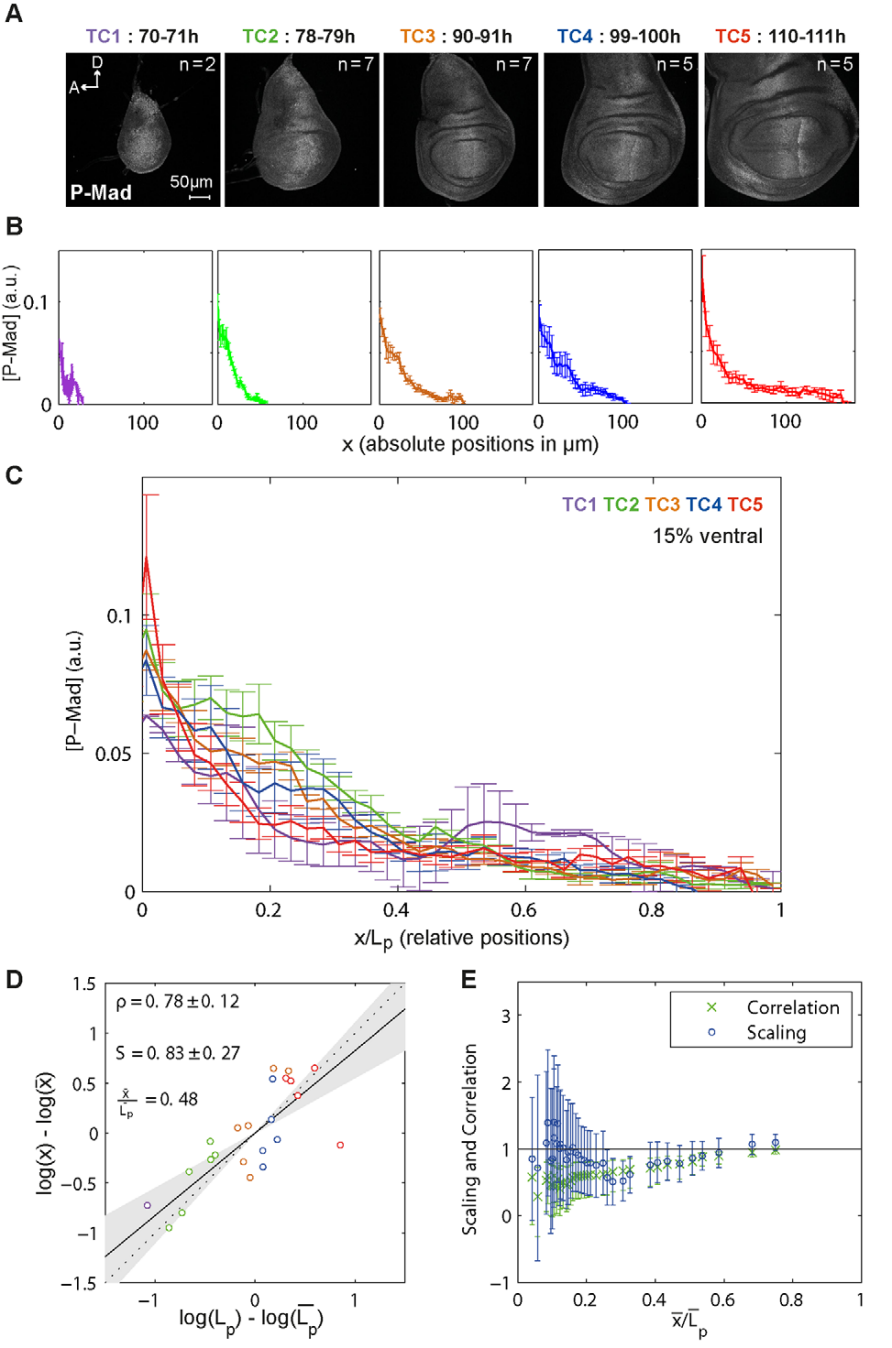
P-Mad gradients expand as the disc grows. (A) Representative wing imaginal discs from each time class (TC) stained for P-Mad antibodies. (B) Average P-Mad profiles at 15% ventral offset. Profiles are shown separately for each TC and in absolute positions. Error bars show the standard error here and in the similar panels of the following figures. (C) Profiles in (B) shown in relative positions. (D) P-Mad scaling corresponding to an average relative position 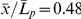. For each disc, log-deviations in the position 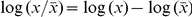 are plotted against log-deviations in the posterior compartment length, 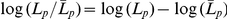, for a given P-Mad threshold concentration. The scaling coefficient S is obtained by linear regression (95% confidence interval on the slope, i.e. on the scaling coefficient, is shown in gray). The dotted line represents perfect scaling (S = 1) here and in subsequent figures. Correlation ρ with its 95% confidence interval is shown. (E) P-Mad scaling (o) and correlation (x) for several threshold concentrations. Error bars represent the 95% confidence intervals.

Before we could ask questions regarding the scaling behavior of Dpp signaling readout during growth of the wing imaginal disc, it was necessary to establish methods to acquire images that can be quantified. We concentrated our analysis on the pouch of the wing imaginal disc, which gives rise to the future wing. To extract the pouch and determine the A/P and D/V compartment boundaries, we co-stained all discs with Wingless (Wg) and Patched (Ptc) antibodies (Figure 1A). Ptc is induced at very early stages and is restricted to the anterior side of the A/P boundary, hence marking the A/P boundary at all the examined stages. Wg expression gets refined later; it outlines the pouch and the D/V boundary starting from 65–70 h into development (Figure S2A). Since we wanted to measure parameters exclusively in the pouch area, the discs from 65-h to 70-h-old larvae were the youngest we included in our analysis. To span the subsequent development of the disc, we subdivided the third instar larval stage into 10 h intervals. In this manner, we defined five time classes (TC) and color-coded them as follows: TC1 in purple: 65–75 h after egg laying (AEL); TC2 in green: 75–85 h AEL; TC3 in orange: 85–95 h AEL; TC4 in blue: 95–105 h AEL; TC5 in red: 105–120 h AEL (Figure 2A). Note that timing refers to hours after egg laying and not to hours after hatching; a larva of 70 h AEL would be 46-h-old after hatching. To minimize variation within a single time class, we collected eggs for 1 h only, and dissected only male larvae to avoid variation due to sexual dimorphism. To minimize errors due to sample handling, we processed all samples in parallel in identical solutions, mounted discs from all TCs on the same slide, and imaged them under identical settings. Further details of sample collection and processing are provided in the Materials and Methods section.

To account for the amplitudes of the protein gradients in our analysis and to test for the hypothesis that downstream target gene domains are defined at constant thresholds of P-Mad and Brk, we treated fluorescence intensities as a measure of protein concentrations. To ascertain that the changes in fluorescent intensities reflected changes in protein concentrations in a linear manner, we imaged fluorescent dyes of known concentrations at the same settings we used for our images and determined the linear range for our imaging conditions. We found that the intensities obtained in our experimental recordings indeed fell into the linear range of our imaging conditions (Figure S2D–E).

We define scaling as the preservation of proportions across growth—i.e. if an expression domain spans 30% of the tissue in a young disc, it should also span 30% in an older and larger disc to achieve perfect scaling. In order to assess scaling *qualitatively*, we compared the protein profiles in relative versus absolute positions. Profiles that scale to some degree look closer together (in other words collapse) when plotted in relative positions compared to when plotted in absolute positions (Figure 1B). We also applied a *quantitative* approach that allows us to assess scaling of a morphogen gradient or a gene expression domain [38]. The degree of scaling is quantified in the form of a scaling coefficient *S*, which equals one when scaling is perfect. Importantly, this measure is position dependent, so that a gradient can scale to varying degrees at different positions in the patterning field (Figure 1C–C″). In contrast to the P-Mad and Brk gradients, the scaling of downstream gene expression domains (Sal, Omb) was measured only at a single position, namely their domain boundary. Fitting a Hill function to each profile returns the position of the domain boundary for that disc; note that in this case, the amplitudes are not informative (Figures 1D, S2C). When the protein profile does not expand sufficiently to compensate for tissue growth, the scaling coefficient obtained is below one (referred to as hypo-scaling). In contrast, when the protein profile expands more than would be needed to compensate growth, a scaling coefficient above one is obtained (referred to as hyper-scaling). This measure of scaling is described in more detail in the Materials and Methods section and Figure 1 provides a schematic step-by-step representation of how we quantified scaling in our data.

### The P-Mad Gradient Expands as the Disc Grows

The first event downstream of Dpp receptor complex activation is the phosphorylation of the signal transducer Mad, which we visualized and quantified with P-Mad antibodies. We analyzed P-Mad gradients in wing imaginal discs from different TCs (Figure 2A). We extracted the P-Mad profiles either exactly along the D/V boundary or with different offsets into the dorsal and ventral compartments within the wing pouch (Figures 1A and S3A). This approach yields a global view of the dynamics of the P-Mad gradients during development (Figure 2B, see Figures S3 and S4 for P-Mad profiles in relative and absolute distances, extracted at several offsets). We observed that the P-Mad levels are significantly suppressed at the D/V boundary in the last TC (TC5; red). Along the D/V axis, the average amplitudes are 25%–30% lower than in the other TCs and the profiles become steeper at this last stage (Figure S3B). However, this effect diminishes gradually away from the D/V boundary, suggesting that it is caused by a factor acting at the D/V boundary (Figure S3). In order to minimize the impact of this effect and of the influence of a secondary Dpp source expressed in the dorsal posterior compartment (red arrows in Figure S1A) [39], we performed our scaling analysis for protein profiles in the ventral compartment with 15% offsets (light purple demarcation in Figure 1A).

For further analyses, we concentrated on the posterior half of the pouch to exclude Dpp secreting anterior cells from the analysis and to avoid complications arising from the modifications of Dpp receptor levels via Hh, which is active only in the anterior compartment [40],[41]. Qualitatively, from TC2 to TC4, the P-Mad gradient expands and adjusts to the disc size, displaying this trend regardless of where it was measured (Figures 2C and S3). Quantitatively, P-Mad shows close to perfect scaling with *S* = 0.83±0.27 at a threshold concentration corresponding approximately to the mid-posterior compartment (x = 0.48 L_p_, where L_p_ stands for the length of the posterior compartment). The individual discs are represented with color-coded circles according to their age (Figure 2D).

In order to obtain a position-dependent picture of scaling, we considered several other protein concentration thresholds and calculated a scaling coefficient at each threshold (Figure 2E). Scaling coefficients (blue circles) and correlation coefficients (green crosses)—informative for the goodness of fit—were plotted as a function of average relative positions, with 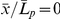 corresponding to the intercept with the A/P axis and 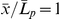 to the end of the pouch for each disc. We found that the P-Mad gradient shows overall very good scaling for 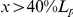 (Figures 2E and S4E–F; closer to the A/P boundary, the error bars are too large for meaningful conclusions). Accordingly, the scaling of a target gene domain that strictly depends on P-Mad levels should follow the same trend. We therefore analyzed known P-Mad targets with this question in mind.

### *brk* Adapts to Disc Size with Increasing Amplitudes

**Figure 3:**
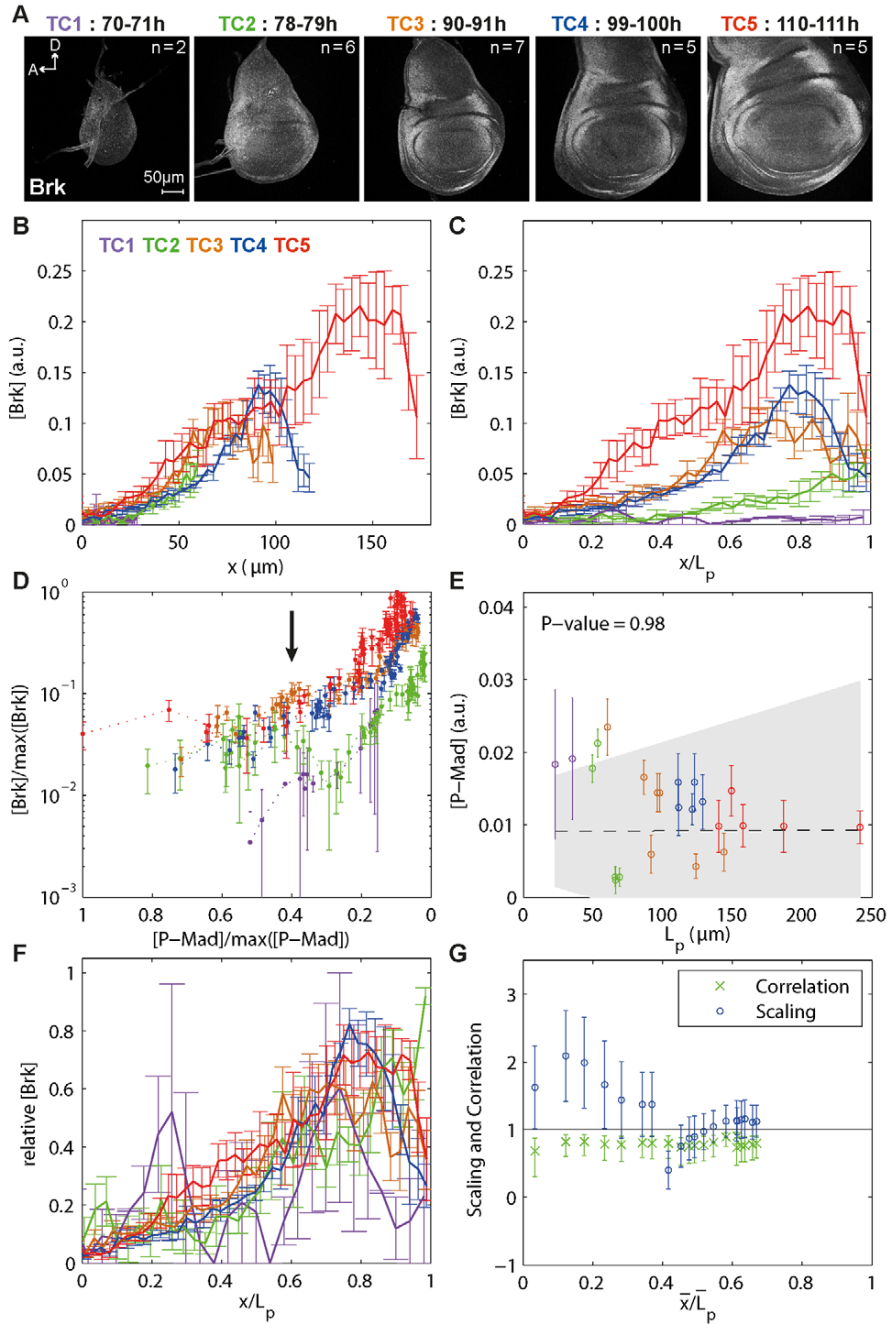
The range of Brk scales with tissue size. (A) Representative images of wing discs for each TC stained with Brk antibodies. (B) Average Brk profiles per TC, measured at 15% ventral offset. (C) Profiles in (B) normalized for L_p_. Note that the Brk concentration at a relative position increases with time. (D) Brk versus P-Mad concentrations for every position in the posterior compartment at 15% ventral offset (P-Mad and Brk from dataset 1 were co-stained). P-Mad and Brk concentrations were normalized to their maximum over all TCs: max ([P-Mad]) = 0.12, max ([Brk]) = 0.22. Arrow points to 40% of max ([P-Mad]). (E) Brk profiles were fitted with a decaying exponential function, yielding a decay length λ_Brk_. For each disc, the average P-Mad concentration at the position x = λ_Brk_ was calculated. The weighted linear regression with 95% confidence interval (gray area) and its *t* test *p* value under the null hypothesis that the slope is equal to zero are shown. (F) Profiles in (B) normalized for maximal intensity and L_p_. (G) Brk scaling (o) and correlation(x) for several threshold concentrations. Error bars represent the 95% confidence intervals.

*brk* is a direct target of Dpp signaling. Its transcription is completely repressed in cells with high levels of Dpp activity, such as the medial cells, and is derepressed to varying levels in response to the decreasing Dpp activity gradient in the lateral parts of the wing disc (Figure S1) [19],[20],[21]. We generated two independent datasets for Brk that we present in two separate figures (Figures 3 and S5). Importantly, both datasets yielded very similar results, demonstrating the reproducibility of our protocol. We examined whether the P-Mad dynamics were mirrored in Brk protein levels. We found that, as the disc grows, the region of maximum Brk expression is found at a further absolute distance from the A/P boundary, consistent with the fact that P-Mad gradients now reach further out and suppress *brk* transcription (Figure 3A–B). Cells expressing highest levels of Brk are found around 80% L_p_ throughout development, suggesting that behind this relative position, P-Mad levels are too low to suppress *brk* transcription (Figures 3C, S5D).

As a result of P-Mad gradients scaling while keeping their amplitudes roughly constant, cells at the same relative position in the disc have very similar P-Mad levels across development. Interestingly, the same cells are subject to increasing levels of Brk: while the magnitude of the increase tends to be smaller away from the D/V boundary, we detected a 10- to 20-fold increase in the average amplitudes in the 40-h interval we studied (Figures 3C and S5A,B,D). We observed a similar trend in the expression levels of Brk with a *brk*-GFP reporter line in which GFP expression is driven by the wing enhancer of *brk* (not shown) [25]. Hence, the changes in Brk protein levels are unlikely to be due to post-transcriptional events.

How are the constant P-Mad levels at a given relative position translated into increasing Brk levels? Since discs were co-stained for Brk and P-Mad, we investigated the relation between Brk concentrations and P-Mad concentrations within each disc. Figure 3D shows the average measured response of P-Mad and Brk within each TC (after normalizing their maximum concentration over all TCs to one). At P-Mad concentrations above 40% of maximum levels, Brk responds similarly to P-Mad at all TCs. However, below this threshold (to the right of the arrow), it appears that Brk can accumulate (Figure 3D). Finally, if we fit decaying exponentials to the Brk expression profiles, the resulting decay lengths correspond roughly to mid-posterior compartment throughout development, a position where P-Mad scales very precisely (Figure 2E). Hence, the length scale of the Brk expression profile corresponds to very similar P-Mad levels across TCs (Figures 3E and S5G). Therefore, the expression pattern of Brk depends on P-Mad while the increase in protein levels cannot be explained with the P-Mad dynamics alone.

Finally, we studied the scaling properties of the Brk profiles. Apart from being non-quantitative, looking at the collapse of the profiles adjusted to compartment size is a good indicator of the level of scaling (Figure 1B). We found that Brk profiles show good scaling only between TC3 and TC4 (Figure 3C, orange and blue profiles). However, when Brk intensities are normalized to their maximum, all profiles collapse rather well (Figures 3F and S5E), and we measured nearly perfect scaling in the lateral part of the pouch (while they seem to hyper-scale more medially), which is in agreement with the measured scaling for P-Mad (Figures 3G and S5F, compare to Figure 2E). Overall, we conclude that the range but not the levels of the Brk gradients scale with the tissue size.

### Dad Expression Incorporates P-Mad and Brk Activities and Shows Position-Dependent Scaling

**Figure 4:**
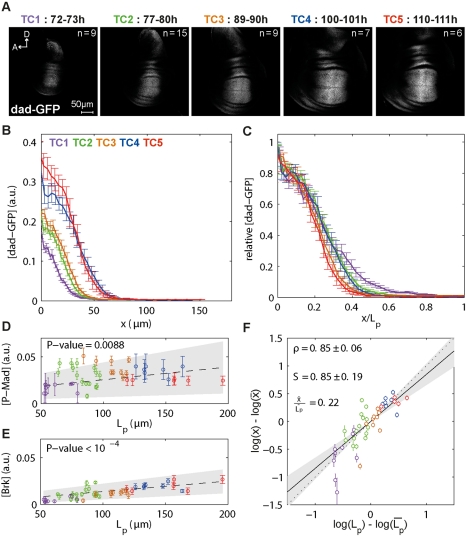
*dad*-GFP displays position-dependent scaling. (A) Representative images of wing discs from larvae carrying the *dad*-GFP transgenes. (B) Average *dad*-GFP profiles per TC at 15% ventral offset. (C) Profiles in (B), normalized for L_p_ and for their amplitudes. (D) dad-GFP profiles were fitted with a Hill function describing the transition point K*_dad_*_-GFP_ of the domain (see Figure S2C). The average P-Mad concentration at the position x = K*_dad_*_-GFP_ against L_p_ for each disc were plotted and the weighted linear regression with 95% confidence interval (gray area) and its *t* test *p* value under the null hypothesis that the slope is equal to zero are shown. (E) Average Brk concentration at the position x = K*_dad_*_-GFP_ plotted against L_p_ for each disc. (F) For each disc, the log-deviations in *dad*-GFP domain boundary position (as defined by the Hill fit; error bars represent the 95% confidence interval of this fit) were plotted as a function of the log-deviations in L_p_. The scaling coefficient S is obtained by weighted linear regression (95% confidence interval in gray). The correlation ρ of the data with 95% confidence interval is shown.

Since there are no antibodies available which recognize Dad, we visualized changes in *dad* expression over time with a *dad*-GFP transgene where the GFP expression is controlled by a 2 kb *dad* enhancer fragment (Figure 4A). This enhancer fragment incorporates positive input from P-Mad as well as negative input from Brk [26],[42]. Hence, *dad*-GFP represents a good tool to monitor combined activity of P-Mad and Brk. While *dad*-GFP forms a gradient reminiscent of P-Mad, it does not tail off as far as P-Mad, presumably because it is sharpened by Brk (Figure 4B) [26]. As a result, while the *dad*-GFP expression pattern can be treated as a gradient (Figure S6H), a Hill function also yields a good fit. The boundary of the *dad-GFP* domain was obtained by fitting a Hill function to each profile and corresponds to x = K*_dad_* (Figure S2C for explanations on the Hill fit). We investigated scaling of the *dad* expression domain along the D/V boundary and with several offsets. Because the *dad-*GFP domain boundary is not straight but contracts at the D/V boundary, especially during the last TCs (Figures 4A and S6A), scaling is quite different depending on where it is measured: the *dad-*GFP domain boundary shows good scaling along the D/V axis (S = 0.94±0.15), at 25% (S = 1.07±0.31) and 15% (S = 0.85±0.19) ventral offsets, while it hypo-scales when measured close to the D/V boundary (S = 0.46±0.17 at 5% dorsal offset where the expression domain is narrowest) (Figures 4F and S6E–G). Therefore, while the *dad* expression domain definitely expands with the growing tissue, it scales to varying degrees at different distances from the D/V boundary.

Similar to Brk levels, *dad*-GFP levels increase with time (Figures 4A–B and S6C). Since *dad* transcription is controlled by both P-Mad and Brk, we asked whether the *dad* domain was specified at constant thresholds of these gradients. We found that levels of both P-Mad and Brk corresponding to the *dad* domain boundary position x = K*_dad_* increase over time (Figure 4D–E). Hence, the Dad domain is not defined at constant P-Mad and Brk concentration thresholds.

### Sal and Omb Domains Scale Well Where They Define Vein Positions

**Figure 5:**
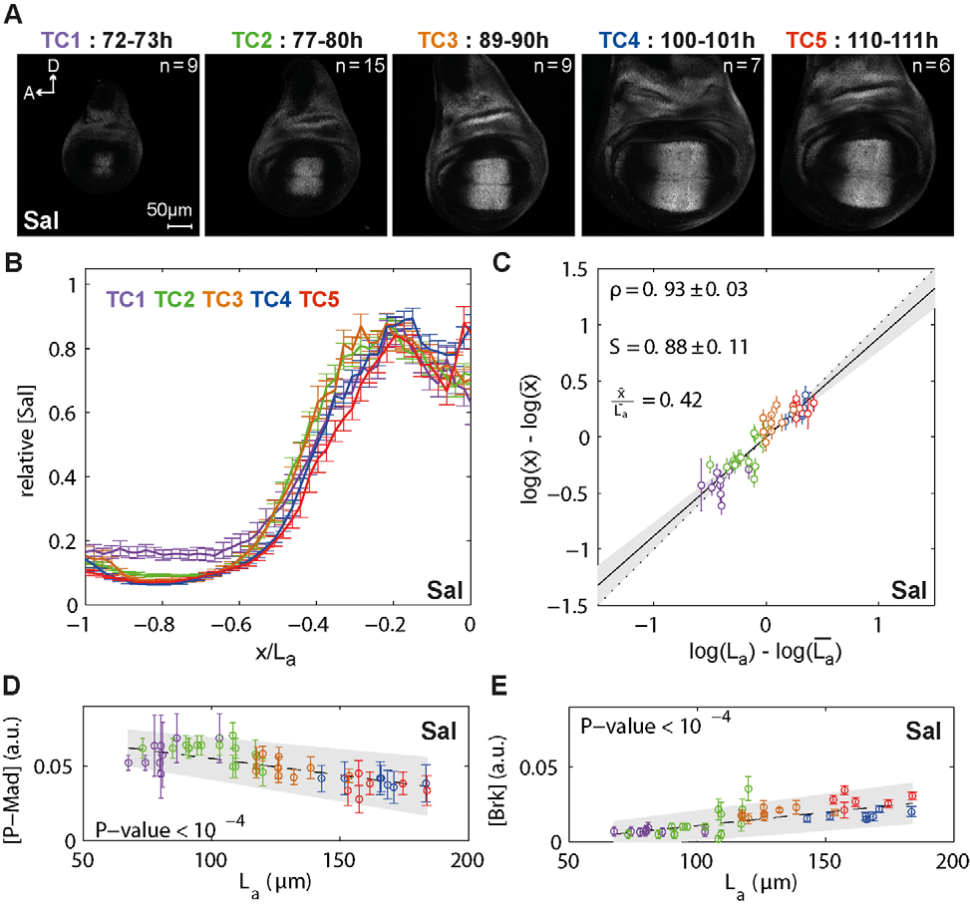
Sal domain scales with tissue size in the anterior compartment. (A) Representative images of Sal antibody stainings at each TC. (B) Average Sal profiles obtained from the individual profiles normalized to their maximum in the anterior compartment at 15% ventral offset, in relative positions. (C) For each disc, the log-deviations in Sal anterior domain boundary position were plotted as a function of the log-deviations in the anterior compartment length L_a_. The scaling coefficient S and the correlation ρ of the data with 95% confidence intervals are shown. (D–E) Average P-Mad (D) and Brk (E) concentrations at the position x = K_Sal_ plotted against L_a_ for each disc. The weighted linear regression with 95% confidence interval (gray area) and its *t* test *p* value under the null hypothesis that the slope is equal to zero are also shown.

**Figure 6:**
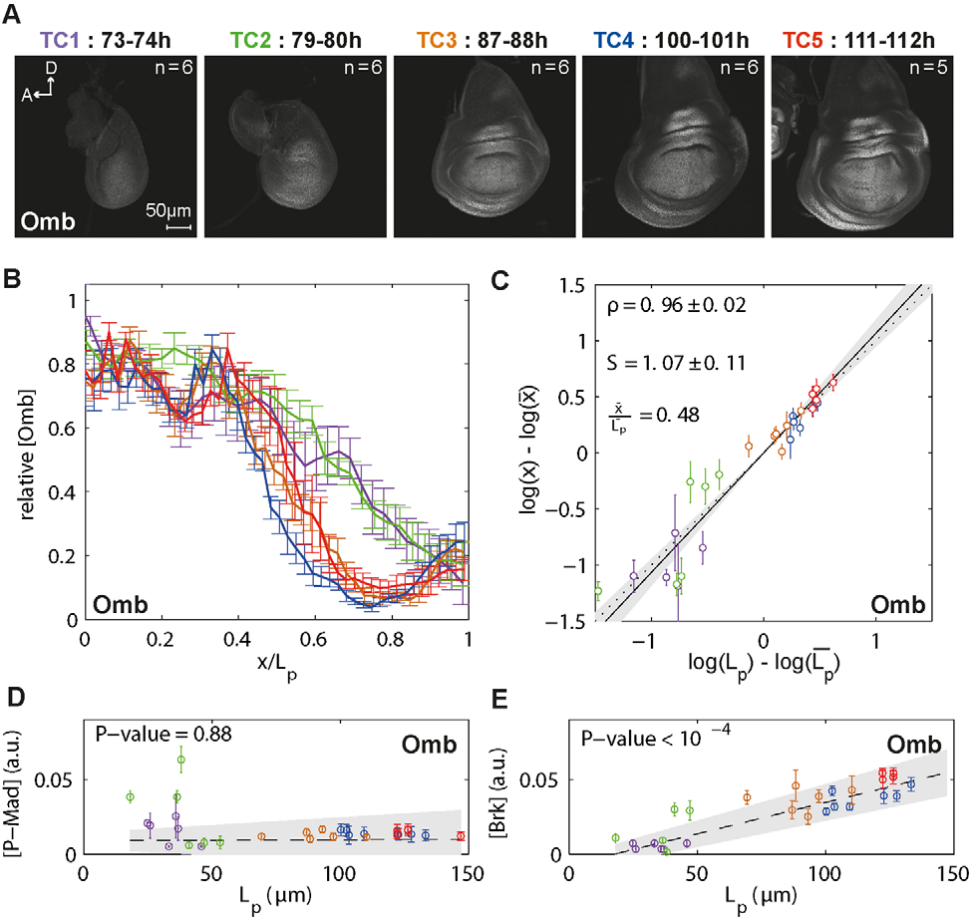
L5 vein position already scale with tissue size in the larval stages. (A) Representative images of Omb antibody stainings at each TC. (B) Average Omb profiles at 15% ventral offset, obtained from the individual profiles normalized to their maximum. (C) For each disc, the log-deviations in Omb posterior domain boundary position were plotted as a function of the log-deviations in the posterior compartment length L_p_. The scaling coefficient S and the correlation ρ of the data with 95% confidence intervals are shown. (D–E) Average P-Mad (D) and Brk (E) concentration at the position x = K_Omb_ plotted against L_p_ for each disc. The weighted linear regression with 95% confidence interval (gray area) and its *t* test *p* value under the null hypothesis that the slope is equal to zero are also shown.

Proper positioning of the veins in the developing wing requires Dpp signaling and is important to ensure adult wing functionality [10],[11],[15]. We asked whether the Sal and Omb domains, informative for positioning veins L2 and L5, respectively, already scale with tissue size during larval stages (Figure S1C). Sal starts to be expressed in the pouch only from the beginning of the third instar stage while Omb is already induced earlier (Figures 5A and 6A). The Sal domain in the anterior compartment spans 40%–45% of the pouch, while it is much narrower in the posterior compartment reaching only up to 15% (Figures 5A–B and S7B,G). Since the vein L2 is located in the anterior compartment, we investigated the scaling properties of Sal in both compartments. Sal profiles expand with the growing disc with increasing amplitudes of roughly 4-fold, and most of this amplitude increase takes place within the first 20 h of the third instar stage (Figures 5A and S7B–C,E). It was previously reported that the Sal domain boundary position correlates with the disc size at the end of development [11]. Consistent with this result, we found that the Sal domain boundary correlates with the lengths of both anterior and posterior compartments throughout development. However, a good correlation is not sufficient to ensure scaling, and indeed we observed that the Sal domain boundary exhibits significant hyper-scaling in the posterior compartment (S = 1.44±0.3, Figure S7G), while it scales well in the anterior compartment (S = 0.88±0.11, Figure 5C). This finding suggests that there might be additional factors at work in the anterior compartment to ensure proper Sal scaling.

The *omb* gene is expressed in a domain larger than Sal, spanning about half the pouch in the posterior compartment (Figure 6A–B), and its domain boundary sharpens during development (Figure S8D). We found that the position of the boundary is well correlated with the length of the posterior compartment and Omb exhibits close to perfect scaling (S = 1.07±0.11 at 15% ventral offset, Figure 6C).

We also investigated whether the boundaries of Sal and Omb expression domains are defined at constant P-Mad and Brk levels. We found that the anterior Sal domain boundary corresponds to decreasing P-Mad levels and increasing Brk levels over time (Figures 5D–E and S7H–I). In the case of Omb, clonal analysis with *brk* loss of function alleles suggests that positioning of the Omb domain boundary strictly depends on Brk activity [19],[43]. In fact, Omb has no direct input from Mad/Medea complexes and is only indirectly activated by Dpp via repression of Brk [14]. In light of these findings, it is surprising that the Omb domain boundary corresponds to similar levels of P-Mad and increasing amounts of Brk during growth (Figure 6D–E). This result suggests that the *dad*, *sal*, and *omb* enhancers become desensitized to Brk as development proceeds.

Overall, we have shown that the P-Mad gradient and the expression domains of its target genes scale rather well with the growing wing disc. Additionally, Teleman et al. found that when the posterior compartment is enlarged or reduced in size via modifications of Insulin signaling activity, the size of the Sal domain adjusts accordingly [44]. These observations suggest that there might be an active mechanism in place to ensure scaling of Dpp activity with tissue size, and raise the question of the identity of the involved players.

### Pentagone Function Is Essential for the Scaling Properties of Dpp Activity Readouts

**Figure 7:**
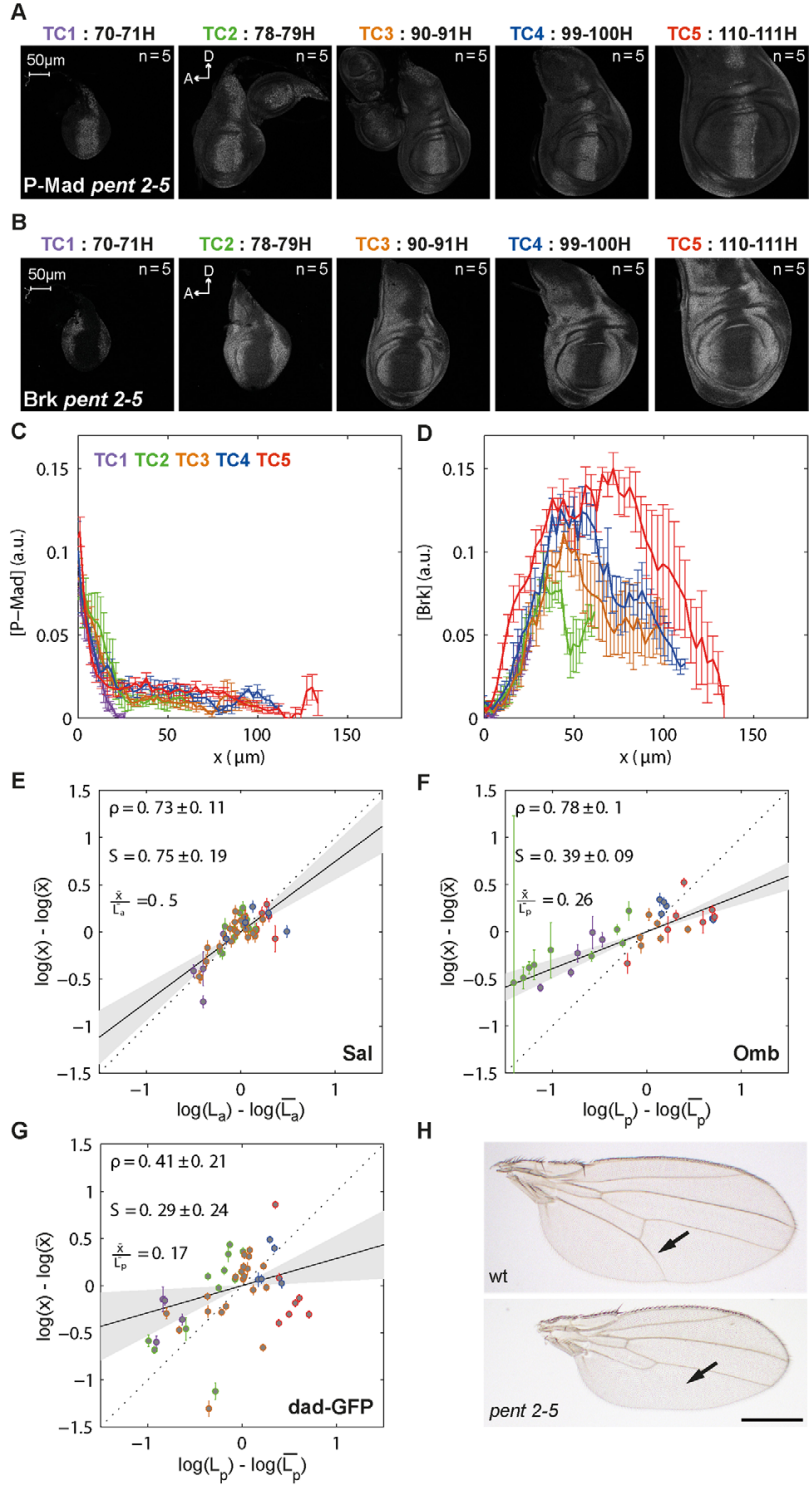
Scaling requires Pentagone function. (A–B) Representative images of P-Mad (A) and Brk (B) antibody stainings at each TC in *pent^2–5^* mutant background. (C–D) Average P-Mad (C) and Brk (D) profiles at 15% ventral offset in *pent^2–5^* display no adaptation to growing disc size. (E–G) Scaling and correlation of anterior Sal (E), posterior Omb (F), and posterior *dad*-GFP (G) domains in *pent^2–5^* mutant background. (H) *Wild-type (wt)* and *pent^2–5^* mutant adult fly wings. Arrows point to the position of L5, which is missing in *pent^2–5^* mutants. The scale bar is 500 µm.

We recently identified a Pent-dependent feedback loop as a major modifier of the Dpp activity gradient [33]. Here we repeated our analyses in a *pent* mutant background using a null allele, *pent^2–5^*, in order to examine its potential involvement in scaling the Dpp activity gradient during disc growth. While Pent function is absolutely essential for proper Dpp gradient formation and maintenance, in its absence flies are semi-viable and overall smaller with wings that lack L5, hence the name of the gene (Figure 7H) [33]. In the absence of *pent*, the defects in the Dpp activity gradient are visible very early on, with P-Mad and Brk forming very steep gradients that resemble sharp domains in all the TCs we examined (Figure 7A–D). P-Mad amplitudes are similar to wild-type levels in *pent* deficient discs and stay rather constant across growth (Figure S9). We observed that the P-Mad domain expands from TC1 to TC2, and after that does not expand any further and hence does not scale with tissue size (Figure 7C, very poor collapse of profiles in relative positions can be seen in Figure S9). Interestingly, none of the D/V related changes that took place in TC5 in the wild-type background were observed in the absence of *pent*; P-Mad levels were not suppressed significantly along the D/V axis and the profiles looked similar at different distances from the boundary, suggesting that Pent is a contributor to this effect (Figure S9). Indeed, Pent binds to Dally [33], which has a role in shaping gradients of both Wg and Dpp [45],[46],[47],[48], and hence the D/V centered effects on the P-Mad profiles we describe here are likely related to this connection.

Are these changes in P-Mad dynamics in *pent* mutants reflected in the expression patterns of its target genes? Repression of Brk via P-Mad does not seem to be affected by the removal of *pent*, as Brk still closely follows P-Mad (Figure 7B,D). In the absence of *pent*, the Brk domain moves more interiorly following the narrower P-Mad domain. Cells with the highest levels of Brk are roughly in the middle or even more proximal, which represents a significant shift compared to being at x = 0.8 L_p_ in wild-type discs (Figures 7D and S10D). Expression of Brk more interiorly in the absence of *pent* is likely to be a major contributor to the growth defects of these mutants as Brk is a well-established growth inhibitor [49],[50]. Consistent with this hypothesis, heterozygosity for *brk* is able to suppress these effects to a large extent (Figure S11). Like in the case of P-Mad, the graded expression pattern of Brk is lost in the absence of *pent*, and the Brk domain does not scale with the tissue size (Figures 7D and S10D).

Next, we asked whether this failure of Dpp activity gradients to adjust to tissue size in *pent* mutants led to narrower expression domains of downstream targets. We found that this was indeed the case, especially in the posterior compartment (Figure S10A–C). In wild-type discs, we observed very good scaling of Sal in the anterior compartment (S = 0.88±0.11 at 15% ventral offset) where it helps to position L2 while it hyper-scales in the posterior compartment. Interestingly, in *pent* mutant discs, scaling of Sal is less affected in the anterior compartment (S = 0.75±0.19 at 15% ventral offset) and the adult flies still have a properly positioned L2 (Figures 7E,H and S10E). Scaling of Omb in the posterior compartment, however, is reduced from almost perfect in wild-type (S = 1.07±0.11 at 15% ventral offset) to nearly lost (S = 0.39±0.09 at 15% ventral offset), while correlations are still very good (Figure 7F). Similarly, scaling of *dad*-GFP along the D/V boundary and with various offsets is greatly reduced in *pent* mutants (Figures 7G and S10A). In the absence of *pent*, the Omb domain boundary defined by the Hill fit shrinks to a quarter of the posterior compartment (x/L_p_ = 0.26). Despite this shrinkage, the Omb domain overlaps significantly with the Brk domain in this background, especially at the end of the third instar stage, a phenomenon not observed to this extent in wild-type discs. A large stripe of cells express both Omb and Brk, raising the possibility that failure to define L5 might be due to this extensive overlap (Figure S11).

We conclude that the adaptation of the Dpp activity gradient to tissue size described in the first part of this study strictly requires Pent function.

## Discussion

### Quantitative Readout of Dpp Activity Dynamics

In this study, we carefully analyzed the dynamics and the scaling properties of the Dpp activity readouts in the growing wing imaginal discs. We discuss our findings with regard to models that were put forward to explain scaling (the expansion-repression model) [34], pattern formation (Figure 1E), and uniform growth [37].

We measured pathway activity using an antibody specific to the phosphorylated form of Mad, and compared the P-Mad levels in space and time with the activity levels of direct target genes, such as *brk*, which plays key roles in both growth and patterning [19],[20],[49],[50]. Transcription of *brk* is directly repressed via P-Mad binding at defined SEs, resulting in inversely graded *brk* expression [23],[25],[51]. Brk is the only known regulator affecting the positioning of the expression boundary of *omb*, while *sal* and *dad* translate input from both P-Mad and Brk into their expression boundaries [14]. We analyzed the dynamics of all of these readouts using antibodies where possible, to avoid potential misinterpretations due to reporter stability.

We found that P-Mad levels scale very well posterior to 0.4 L_p_ with the exception of TC5 profiles near the D/V boundary. Previous studies that examined P-Mad scaling reached contradictory conclusions: the P-Mad gradients in late stage discs were reported to correlate with tissue size in a previous study [44] and to have no correlation in another [11]. Similarly, examination of P-Mad gradients across discs of different sizes led to the conclusion that the gradient did not expand [52], but more recently the P-Mad gradient was shown to scale with tissue size [37]. We believe that most of the confusion can be attributed to different profile extraction protocols as well as to the use of various definitions of scaling, as discussed below.

Since P-Mad is an early signature of the activation of the Dpp signaling pathway, we wanted to find out how its scaling properties translate to its immediate key target, the *brk* gene. In addition to *brk* being directly repressed by P-Mad, the Brk protein itself is necessary for graded *brk* transcription [53]. We found that the range of Brk expression strictly follows P-Mad in both wild-type and *pent* mutant discs. Similar to what we observe for P-Mad, Brk also shows very good scaling for positions posterior to 0.4 L_p_. By contrast, levels of Brk increase steadily as the discs grow and cannot be explained by P-Mad dynamics alone. This increase in Brk levels could be due to the build-up of the unknown activator of *brk* transcription or, alternatively, the SEs in *brk* could become desensitized to the repressive input of P-Mad. Regardless of the cause of the increase, cells at a given relative position experience increasing levels of the Brk repressor over time.

### Different Definitions of Scaling

Traditionally, Dpp and P-Mad gradients have been described by a decaying exponential with characteristic decay length λ [11],[37],[54]. This decay length is different for each profile and corresponds to the position at which the protein levels have decreased by a factor *e*. The correlation between the decay length and the tissue size has been used as a proxy for scaling, e.g. in the work of Bollenbach et al., which found no significant scaling for the Dpp and P-Mad profiles at the end of 3^rd^ instar stage (note that they used the length of the pouch along the A/P boundary as a measure of tissue size, [11]). Similarly, the width of the P-Mad profile has been used to characterize the spread of the gradient and it was concluded that the width of the P-Mad profile is constant during growth [55]. In contrast, we detected that the P-Mad profile expands as the tissue grows. This discrepancy may be due to the fact that Hufnagel et al. lacked TC3 and TC4 in their sample collection, the period where the P-Mad gradient expands before contracting again when measured close to the D/V boundary (Figure S4B, compare red and green). Thus, possibly the measurements of the P-Mad profiles were done in the vicinity of the D/V boundary, where at TC5 P-Mad has a sharp profile reminiscent of 30 h younger discs. Hence, the choice of position can significantly alter the final interpretations of the data.

Consistent with our results, Wartlick et al. recently showed that the decay lengths of Dpp-GFP, P-Mad, *brk*-GFP, and *dad*-RFP do correlate with the length and the area of the posterior compartment during tissue growth [37]. Importantly, they also assessed scaling *qualitatively* in the whole field by looking at the collapse of the profiles in relative positions and normalized intensities (Figure 1B). This method has two advantages: it does not require any fitting of the profiles, and it shows scaling at all positions and not just at the characteristic decay length position (i.e. *x* = λ).

In our work, we used the raw intensity measurements without fitting any function to the corresponding profiles, since the exponential is not the best fit at all time classes. Similarly, we wanted to assess scaling in the whole field and not just at one characteristic position. To this end, in addition to looking at the collapse of the profiles, we used our measure of scaling [38], which gives a *quantitative* measure for each position in the tissue (Figure 1C). Lastly, we would like to emphasize that we did not normalize the P-Mad intensities before measuring scaling coefficients as the absolute protein levels are crucial for signal interpretation in the simplest scenario. In the case of Brk, however, whose amplitudes increase steadily, scaling only emerged after normalizing the intensities.

For comparison, we also show in Figure S12 the ratios of the decay lengths over the pouch sizes for all the genes we investigated. Since the more downstream target genes were better fitted with a Hill function, we also report the ratio of the corresponding transition points as a function of pouch size for those genes. In agreement with Wartlick et al, we find that P-Mad and Brk decay lengths correlate well with tissue size, with λ_P-Mad_ = 0.21 L_p_ and λ_Brk_ = 0.18 L_p_. We note that our estimate of λ_P-Mad_ is smaller than the previously reported value (0.34 L_p_), likely due to the fact that we measure tissue size along the D/V boundary, from the intersection of the A/P to the limits of the pouch, as opposed to the length of the posterior compartment at its widest position. Our measure of λ_Brk_ is very similar to that of Wartlick et al. Hence, considering that we have a larger value for the tissue size, Brk protein must form a gradient with a larger decay length than the *brk*-GFP reporter that was used by Wartlick et al. [37].

### Is Pentagone the “Expander” of the Dpp Gradient?

A recent mathematical model termed “expansion-repression integral feedback control” suggests that scaling can emerge as a natural consequence of a feedback loop [34]. The hypothetical “expander” molecule facilitates the spread of the morphogen and in turn is repressed by it; scaling is achieved given that the expander is stable and diffusible. The known properties of Pent fit the requirements of this hypothetical agent: Pent is secreted, required for Dpp spreading, and *pent* transcription is directly inhibited by Dpp signaling. However, we do not know how stable Pent is, and *pent* transcription is never abolished in the entire field in which the gradient acts during larval stages. To test whether Pent could be a key player involved in scaling of Dpp activity during disc growth, we repeated our analyses at all time points in the absence of Pent. We found that the P-Mad and Brk gradients indeed fail to scale with the tissue size in this mutant background. Scaling of *dad*-GFP and Omb are also strongly affected, while Sal still exhibits some degree of Pent-independent scaling in the anterior compartment. Importantly, while the function of Pent is essential for proper scaling of the Dpp activity gradient, we note that Pent alone cannot account for the observed selective scaling of Omb and Sal domain boundaries. Scaling of these target genes specifically in those regions in which they have a patterning function points to the involvement of additional players, which will be the subject of future research. Hence, our findings strongly suggest that Pent is a very good candidate to be the expander in the “expansion-repression integral feedback control” model and therefore provide the first mechanistic insights into the question of scaling in wing patterning. The exact biochemical functions of Pent have to be determined in order to get a more mechanical view of gradient scaling in the developing wing imaginal disc.

### Domain Boundaries and the Interpretation of the Activity Gradients

More than 40 y ago, Lewis Wolpert proposed the French flag model to explain pattern formation by morphogens (Figure 1E) [35]. Here we tested whether the activity gradients downstream of Dpp, namely P-Mad and Brk, are read out by their target genes at constant concentration thresholds. Thus, we measured average P-Mad and Brk concentrations at Dad, Omb, and Sal expression boundaries across development. We found that the amount of P-Mad at these boundaries slightly increased (Dad), slightly decreased (Sal), or was constant throughout development (Omb). Among these three targets, the Omb domain is the widest and it corresponds to a region where the P-Mad gradients scale perfectly; as a result, P-Mad levels fluctuate very little at the Omb domain boundary. Interestingly, the domain boundary of Omb is thought to solely depend on Brk and hence constant P-Mad levels might be a mere coincidence. Remarkably, all the target genes we considered respond to significantly increasing levels of Brk, suggesting that the target genes desensitize to Brk over time, so that more and more Brk can be tolerated at the domain boundary. Alternatively, if we consider that the domain boundaries of *dad*-GFP and Sal do not respond to constant P-Mad levels either (Figures 4D and 5D), another explanation could be that Brk and P-Mad signals are combined in a non-additive fashion in order to define the boundary position of the target genes. Following this assumption, we looked for a simple combination of these signals that is constant at the target gene domain boundary for all TCs. For *dad*-GFP in the posterior compartment, the ratio P-Mad^2^/Brk is constant at the domain boundary (*t* test *p* value = 0.13 under the null hypothesis that the slope is equal to zero; in *pent^2–5^*, *t* test *p* value = 0.88), while for Sal in the anterior compartment, the multiplicative combination P-Mad^5^*Brk^4^ is constant (*t* test *p* value = 0.42; in *pent^2–5^*, *t* test *p* value = 0.91). We propose that Brk and the unknown activator of Omb could be similarly combined in order to determine the Omb domain boundary.

### Implications of Our Findings for a New Growth Control Model

We used our data to further test a model that was recently proposed to explain the uniform growth in the wing imaginal disc [37]. The model poses that the temporal changes in Dpp signaling levels drive tissue growth; cells divide when they experience a relative increase of 50% in the levels of Dpp signaling. Since it is the relative differences and not the absolute amount of Dpp signal that regulate cell divisions, the model can account for the uniform growth of the wing disc. Since the relative increase in Dpp activity slows down, the cell cycles lengthen as the disc grows. Growth stops when the cell division time exceeds 30 h. The model of Wartlick et al. is based on the finding that Dpp activity scales with tissue size and that cells at a given relative position experience increasing levels of Dpp signaling over time. In contrast, we do not observe a general temporal increase in the level of Dpp signaling at a given relative position in our study. P-Mad is the most upstream and the most dynamic readout available for the activity of the Dpp pathway and we find that the relative increase in P-Mad levels throughout development is not significantly different from zero at most relative positions (in –A′, almost all the error bars (95% confidence interval) cross the value Δc/c = 0). Why is the increase in Dpp-GFP levels not reflected in P-Mad levels? A potential explanation for this might be that the observed accumulation of Dpp-GFP was due to the stability and accumulation of Gal4 since Dpp-GFP was under UAS control [37]. The authors showed that the half-life of the Dpp-GFP fusion protein is only 20 min, but the Gal4 stability was not considered. Alternatively, the system could get desensitized over time and more and more Dpp would be required to lead to similar P-Mad levels. Finally, increases in Dad levels could counteract the increase in Dpp levels, since Dad is an inhibitory Smad [14],[56].

Wartlick et al. monitored Dpp signaling levels using a *dad*-RFP reporter and found a 5-fold increase in the course of 36 h [37]. In our analysis, we used a similar tool, *dad*-GFP, and failed to fully reproduce their results. Though we also find that *dad*-GFP scales with tissue size, its levels increase merely 2-fold over 40 h, and this increase takes place only in the medial 25% region of the disc, while cells in the lateral part experience a decrease in *dad*-GFP levels (Figure S13C–C′). This disparity in the fold increases is likely due to the higher stability of RFP [57] since the enhancer used, to our knowledge, is identical in both studies. Additionally, we found that the levels of Brk, another direct target of the pathway with a very well-established role in suppressing growth in lateral regions, increase in average 4-fold in the interval studied, an observation not reported by Wartlick et al. (Figure S13B–B′). In the lateral areas, increase in Dpp activity (if present) is below detection levels and would be opposed by increasing Brk levels. Importantly, increasing Brk levels, if they were to depend solely on Dpp, would suggest decreasing Dpp activity in lateral areas as Brk expression is directly suppressed by Dpp activity. Hence, our data raise serious questions about the validity of this uniform growth model, especially in the lateral regions of the pouch. We favor an alternative model that does not rely on Dpp activity alone to explain uniform growth in the wing disc [58].

## Materials and Methods

### Sample Collection

Flies were constantly kept in a 26°C incubator and the eggs were collected on grape juice plates. It is known that the females can keep the fertilized eggs for up to 8 h, so a freshly laid egg can be anywhere between minutes to 8 h old. We circumvented this problem by treating flies with CO_2_ prior to collection, which is thought to relax the muscles and facilitate the deposition of old eggs. This first collection was discarded and the flies were transferred to a clean collection chamber. Additionally, as sexual dimorphism exerts itself early on, only male larvae were included in our analysis where possible. Indeed, male flies are comparatively smaller than female flies and including both sexes could bias our scaling results during wing imaginal disc growth. Male larvae were positively selected for by the presence of a clear, oval genital disc, which is clearly visible starting from 80 h AEL. Therefore, our 70 h collections had both male and female larvae. We observed that 70 h AEL corresponds to the beginning of the third instar stage at 26 °C as hatching larvae were frequently encountered. Dissected larvae were fixed immediately, washed, and stored at 4°C. Once all time classes were obtained (usually within 2 d), all samples were processed for antibody staining in parallel using identical solutions.

### Immunostainings, Image Acquisition

Larvae of different time classes (TC1: 65–75 h AEL, TC2: 75–85 h AEL, TC3: 85–95 h AEL, TC4: 95–105 h AEL, TC5: 105–120 h AEL) were transferred into cold fixative (4% pfa in PBS, pH = 7) and fixed for 25 min at room temperature on a rotator. Following extensive washes in PBT (PBS+0.03% TritonX), the discs were blocked in PBTN (PBT+2% Normal Donkey Serum, Jackson Immuno Research Laboratories) for 1 h at 4°C on a rotator, and incubated with primary antibodies overnight at 4°C. The discs were washed several times with cold PBT and incubated in secondary antibodies for 2 h at room temperature on a rotator. After another round of washes with PBT, the excess fluid was removed and replaced with Vectashield mounting media (Vector Labs). All discs from a dataset (i.e. all 5 TCs) were mounted on the same slide to reduce potential variation in thickness between the slide and the coverslip across different samples. Brain discs were used as spacers. All discs from a dataset were imaged under identical microscopy settings using a Leica SP5 confocal microscope (1 µm thick sections).

### Antibodies and dad-GFP

Rb-α-P-Mad (1∶1,500, Ed Laufer, [41],[59]); rb-α-Sal (1∶40, Reinhard Schuh, [60]); rb-α-Omb (1∶1,200, Gert O. Pflugfelder, [61]); m-α-Wg (a.k.a. 4D4, 1∶120, DSHB, University of Iowa); m-α-Ptc (a.k.a. Apa1, 1∶600, DSHB, University of Iowa); gp-α-Brk (1∶1,000, Gines Morata). All secondary antibodies were used in 1∶1,000 dilutions and were from the AlexaFluor series of Invitrogen. *dad*-GFP transgenic flies were described in [42].

### Image Processing

After the image acquisition, we manually selected by visual inspection four consecutive slices above and below the brightest slice from each stack and performed a mean projection of these nine slices. Using a reduced number of slices and performing the mean projection allowed us to reduce the noise as well as avoid the signal from the peripodial membrane. Indeed, we made sure that these nine slices contained signal from the columnar cells of the pouch only. We then manually contoured the inner pouch boundary as well as the anterior-posterior (A/P) and dorsal-ventral (D/V) boundaries, as marked by the Wg and Ptc stainings. All discs were rotated to have anterior to the left and dorsal upwards orientation. The remaining analyses were applied solely to the pouch. We extracted the profiles along the D/V boundary, since it is a natural coordinate in the wing pouch, or parallel to it with a small offset of 5% of the height of the pouch into the dorsal compartment to avoid potential interference with Wg, which is expressed at the D/V boundary. We repeated our analysis also with 15% and 25% offsets into the ventral compartment. Note that since the D/V boundary is not a thin line but a stripe, we applied mean filtering with a rectangular sliding window of fixed size (20×3) pixels (height×width) to smoothen the images of (1024×1024) pixels before further analyses. Also, because we used nuclear markers, the 1 d extracted profiles looked very rugged and we therefore applied Gaussian filtering before quantifying scaling. We assumed that the changes in cell density are negligible.

### Quantifying Scaling

We aimed to quantify scaling of Dpp target genes. In a previous work [38], we defined scaling as the relative response in gene expression domain position 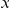 due to variations in tissue size *L*:
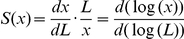
Here perfect scaling corresponds to a scaling coefficient *S* that equals to one, while a scaling coefficient below one indicates hypo-scaling, and a scaling coefficient greater than one corresponds to hyper-scaling. We emphasize that perfect correlation is not equivalent to perfect scaling, since it only guarantees a strictly linear response of the domain position with tissue size, but not the preservation of proportions. Thus, correlations are not informative on whether the shift in domain boundary position during growth is adequate, or if a specific domain position tends to hyper- or hypo-scale [38].

In our analysis, we concentrated on the posterior compartment and used the length of the posterior compartment (L_p_) as a measure of the tissue size. L_p_ is indeed a good measure of tissue size as it changes proportionally with the square-root of the area of the posterior compartment (Figure S2B). Among the Dpp activity readouts we examined, there are proteins that form gradients (P-Mad, Brk, *dad*-GFP) as well as genes that are expressed in rather sharp domains where the boundary position is likely to matter more than the amplitude of the signal (Omb, Sal, *dad*-GFP). We analyzed the scaling properties of *dad* both as a gradient and as a domain as it can be defined either way. In the case of sharp domains, we extracted the protein expression profiles (as deduced from the fluorescent intensities) for each gene of interest in discs of different ages and asked whether the position of the expression domain boundary in the posterior compartment scales with the compartment length (Figure 1D–D′). The boundary of the domain was obtained by fitting a Hill function (with free exponent) to each profile (Figure S2C). We then estimated scaling by weighted linear regression (the weights *w* on the domain boundaries come from the fitting procedure):
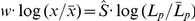
Note that the linear regression assumes that 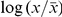 and 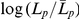 are correlated (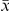 and 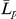 are the average domain boundary position and the average posterior compartment length, respectively). Note that for small deviations 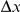 and 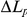 from the mean in boundary position and tissue size we can approximate 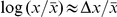 and 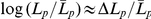 [38].

For assessing the scaling properties of P-mad, we took into account its amplitude, assuming that the absolute concentrations are important for the signal interpretation by the target genes. Moreover, we wanted to characterize scaling over the whole field where the protein is expressed and not just at one particular position, because the gradient can scale differently across positions. We therefore considered several thresholds of protein concentration readout. Note that while an exponential function can provide a reasonable fit to P-Mad and Brk profiles away from their source, we did our calculations without fitting any specific curve to the profiles. For each threshold, we plotted the corresponding positions against the lengths of the posterior compartments for our collection of discs (Figure 1C–C′). Thus, we got a scaling coefficient at each threshold, which we can then associate to the position 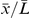. This way, we obtain a scaling coefficient for several relative positions in the patterning field (Figure 1C″).

### Linear Regressions and Statistical Tests

All the linear regressions that we perform are weighted if the data points on the plot have an error bar (weight = 1/error^2^). The gray area represents the 95% confidence interval on the linear regression, that we approximate using the estimated standard error *ste* on the parameter *s*: upper 95% confidence interval = s+1.96*ste, lower 95% confidence interval = s−1.96*ste. Note that in the scaling plots (e.g. Figure 2D), the data points are centered around (0,0), so that the regression line must pass through (0,0). In that case, the gray area represents the 95% confidence interval on the slope only, which is the scaling coefficient, without including that of the intercept. This is why the gray area narrows at (0,0).

All the *p* values are meant to assess whether the slope of the linear regression is significantly different from zero. Thus, we perform a *t* test under the null hypothesis that the slope is equal to zero. In the case where the *p* value is below 0.05, we reject this null hypothesis.

## Supporting Information

Figure S1 Dpp signal transduction and vein formation. (A) Representative images of expression patterns referred to in this study. Red arrows point to the effect of posterior Dpp source on various target gene expression patterns. (B) In the medial cells (left), type I (Thickveins-Tkv) and type II (Punt) receptors—both Ser/Thr kinases—form heterodimers upon Dpp binding allowing constitutively active Punt to phosphorylate and activate Tkv, which in turn phosphorylates the Mad proteins (Receptor-Smad). P-Mad molecules form complexes with Medea (co-Smad) and translocate into the nucleus where they can both activate as well as inhibit transcription of target genes with the help of co-factors. P-Mad/Medea/Schnurri complex represses transcription of *brk* and *pent* via binding to the so-called silencer elements (SEs); hence *brk* and *pent* can only be expressed in the lateral cells. Transcription of *sal* and *dad* are positively regulated by the P-Mad/Medea complexes. Brk is the default repressor of Dpp target genes, and its removal results in derepression of *omb* transcription. In the absence of Dpp signal (right), Brk and Pent are expressed at high levels, and Brk keeps *sal*, *dad*, and *omb* off. Pent is secreted and helps movement of Dpp laterally via binding to the HSPG Dally. In between these two extremes, cells read both P-Mad and Brk gradients, and the sensitivity of enhancers to these two factors as well as others determine their response. Modified from [14], figure template courtesy of Alex Weiss. (C) Third instar wing imaginal discs stained for Blistered (Bs) (red) and Sal (green) on the left and for Bs (red), Omb (green), and Brk (blue) on the right. Bs expression is suppressed in the future veins. The middle panel shows the vein positions in a third instar disc and an adult wing. L2, marked with white tracing on the left, is formed within the anterior edge of the Sal/Salr expression domain, overlapping with very low Sal/Salr levels [28]. The L5 primordium, marked with white tracing on the right, forms within the posterior edge of the Omb domain adjacent to cells expressing high levels of Brk [29].
(TIF)

Figure S2 Methods. (A) Discs of varying ages stained with Wg and Ptc antibodies. Wg staining gets refined by 71–72 h AEL. (B) The posterior compartment length measured along the D/V axis (L_p_) correlates well with the square root of the posterior compartment area (area_p_) both in wt (black) and *pent^2–5^* mutant discs (red). Each dot represents a disc. (C) The Hill function used to fit the gene expression domains returns four parameters: the amplitude Amp, the spread of the domain K, the sharpness of the domain boundary n, and a constant offset c. (D–E) Linear range imaging for P-Mad/Brk dataset 1 (D) and Omb/Brk dataset 2 (E). Several dilutions of the secondary antibodies Alexa 488 (green) and Alexa 568 (red) yield fluorescent intensities that are proportional to their concentrations under our imaging conditions. Mean intensities in the whole field and the standard deviations were obtained using the Histogram function in ImageJ. We measured background by imaging an empty slide and subtracted this value. Linear regressions are indicated with dotted lines.
(PDF)

Figure S3 P-Mad is repressed along the D/V boundary at the end of third instar. (A) The dashed yellow lines outline the pouch as well as the A/P and the D/V compartment boundaries, as defined by Wg and Ptc stainings in red. P-Mad profiles were extracted along the D/V and with 5% (yellow), 15% (purple), 25% (orange) offsets from it. (B–F) P-Mad profiles averaged per TC in relative positions along the D/V (B), with 5% offset into the dorsal compartment (D), and with 5% (C), 15% (E), and 25% (F) offsets into the ventral compartment. Positions in the posterior compartment are normalized relative to the posterior compartment length L_p_, while positions in the anterior compartment are normalized relative to the anterior compartment length L_a_.
(PDF)

Figure S4 P-Mad profiles and amplitudes at various positions. (A–D) P-Mad profiles averaged per TC along the D/V (A), and with 5% dorsal (B), 15% ventral (C), 25% ventral (D) offsets. (A′–D′) The amplitude of the P-Mad profile (i.e. the concentration at A/P compartment boundary, x = 0) plotted versus the posterior compartment length. Each dot represents a disc and is color-coded according to its age. The linear regression with 95% confidence interval (gray area) and its *t* test *p* value under the null hypothesis that the slope is equal to zero are shown. (E–F) P-Mad scaling (o) and correlation (x) for several threshold concentrations using the P-Mad profiles that were extracted with 25% ventral offset (E) and 5% dorsal offset (F). Error bars represent the 95% confidence intervals, obtained from the linear regressions in the case of scaling.
(PDF)

Figure S5 A second dataset for Brk. (A–B) The amplitudes of the Brk profiles (i.e. the peak concentration in the lateral region) at 15% ventral offset versus the posterior compartment length for dataset 1 (A) and dataset 2 (B). Taking the extremes, the ratio between the extreme values of the Brk amplitudes (Amp) are max(Amp)/min(Amp) = 48.7 for dataset 1 and max(Amp)/min(Amp) = 48.3 for dataset 2, respectively. (C) Brk profiles averaged per TC with 15% ventral offset (dataset 2). (D) Profiles in (C) in relative positions. (E) Profiles in (D) with normalized amplitudes. (F) Brk scaling for several threshold concentrations. Error bars represent the 95% confidence intervals. To compute scaling at each position, the Brk profiles with normalized amplitudes were used (dataset 2). (G) Brk profiles were fitted with a decaying exponential function (dataset 2) to obtain the decay length λ_Brk_ of the profile. For each disc, the average P-Mad concentration at the position x = λ_Brk_ was plotted against the L_p_ of the disc. The weighted linear regression with 95% confidence interval (gray area) and its *t* test *p* value under the null hypothesis that the slope is equal to zero are shown.
(PDF)

Figure S6 *dad*-GFP shows position-dependent scaling. (A) For each time class, a representative *dad*-GFP contour plot is shown. Lower concentrations are in light blue, higher concentrations in pink. (B) *dad*-GFP profiles averaged per TC in relative positions, at 15% ventral offset. (C) The amplitudes of the *dad*-GFP profiles (i.e. the concentration at A/P compartment boundary, x = 0) plotted against the L_p_ for each disc, at 15% ventral offset. (D) *dad*-GFP domain boundary at 15% ventral offset gets slightly sharper across growth. Error bars represent 95% confidence intervals from the Hill fits (see also Figure S2C). The weighted linear regression with 95% confidence interval (gray area) and its *t* test *p* value under the null hypothesis that the slope is equal to zero are shown. (E–G) Scaling and correlation of *dad*-GFP domain boundary along the D/V (E), and with 5% dorsal (F), 25% ventral (G) offsets. (H) Scaling of *dad*-GFP for several threshold concentrations when treated as a gradient. Error bars represent the 95% confidence intervals, obtained from the linear regressions in the case of scaling. Consistent with the profiles in (B), positions anterior to ∼0.3 L_p_ show hyper-scaling due to the increasing dad-GFP levels, while positions posterior to it show hypo-scaling.
(PDF)

Figure S7 Sal amplitudes increase over time and the posterior Sal domain hyper-scales. (A) Representative Sal contour plots for each TC. Lower concentrations are in light blue, higher concentrations in pink. (B) Sal profiles averaged per TC at 15% ventral offset. (C, E) Sal amplitudes (i.e. the maximum concentration in the vicinity of the A/P compartment boundary at x = 0) for each disc at 15% ventral offset versus the anterior (C) and posterior (E) compartment lengths. (D, F) Sharpness of the Sal domain boundary (n) in the anterior (D) and the posterior (F) compartments plotted against tissue size. (G) Scaling and correlation of the Sal domain boundary in the posterior compartment at 15% ventral offset. (H–I) The average P-Mad (H) and Brk (I) concentrations at the position x = K_Sal_P_ (Sal boundary in the posterior compartment) were plotted against the L_p_ for each disc. The weighted linear regression with 95% confidence interval (gray area) and its *t* test *p* value under the null hypothesis that the slope is equal to zero are also shown.
(PDF)

Figure S8 Omb domain scales with tissue size. (A) Representative Omb contour plots for each TC. Lower concentrations are in light blue, higher concentrations in pink. (B) Omb profiles averaged per TC at 15% ventral offset. (C) Omb amplitudes (i.e. the concentration at A/P compartment boundary, x = 0) versus the posterior compartment length for each disc at 15% ventral offset. The linear regression with 95% confidence interval (gray area) and its *t* test *p* value under the null hypothesis that the slope is equal to zero are also shown. (D) Sharpness of the Omb domain boundary. The weighted linear regression with 95% confidence interval (gray area) and its *t* test *p* value under the null hypothesis that the slope is equal to zero are also shown.
(PDF)

Figure S9 Lack of size adaptation of P-Mad profiles in *pent^2–5^*. (A–D) P-Mad profiles in *pent^2–5^* mutant background are shown in relative positions and averaged per TC along the D/V (A), and with 5% dorsal (B), 15% ventral (C), 25% ventral (D) offsets. Positions in the posterior compartment are normalized relative to the posterior compartment length L_p_, while positions in the anterior compartment are normalized relative to the anterior compartment length L_a_. (A′–D′) The amplitude of the P-Mad profile in *pent^2–5^* mutant background (i.e. the concentration at A/P compartment boundary, x = 0) was plotted versus the posterior compartment length for each disc. The linear regression with 95% confidence interval (gray area) and its *t* test *p* value under the null hypothesis that the slope is equal to zero are also shown.
(PDF)

Figure S10 Expression patterns of target genes in *pent^2–5^*. (A–C) Representative images of *dad*-GFP (A), Sal (B), and Omb (C) in *pent^2–5^* mutant background. (D) Brk profiles averaged per TC in relative positions at 15% ventral offset in *pent^2–5^* background. Brk levels still increase in *pent* mutants and the profiles move relatively inwards as the discs grow. (E) Posterior Sal domain boundary does not scale in *pent^2–5^* discs.
(PDF)

Figure S11 Omb and Brk domains overlap extensively in *pent^2–5^*, and heterozygosity for *brk* rescues growth defects of *pent* mutants. (A–A″) wt (B–B″) *pent^2–5^* (C–C″) *brk^XA^+/−*; *pent^2–5^*. (A, B, C) Third instar wing imaginal discs stained for Omb (red) and Brk (green). (A′, B′, C′) Omb channel only (gray). (A″, B″, C″) Representative wings from female flies of corresponding genotypes. Heterozygosity for *brk* rescues growth defects of *pent* mutants to a large extent. The slight rescue of L5 shown in C″ is highly variable and represents an average wing; some flies have an almost complete rescue of L5 while others display no rescue.
(PDF)

Figure S12 Decay length λ correlates with tissue size. (A) P-Mad, Brk, and *dad*-GFP profiles were fitted with an exponential and the resulting decay lengths (λ) were plotted as a function of the posterior compartment length L_p_. The text in the plot shows the average λ/L_p_ ratio with its standard error. Relationships obtained from the linear regression are displayed in the boxes on the right. Note that L_p_ is measured along the D/V compartment boundary. (B) Same as (A), but the *dad*-GFP, Sal, and Omb profiles were fitted with a Hill function instead of an exponential.
(PDF)

Figure S13Changes in P-Mad, Brk, and *dad*-GFP levels at relative positions. (A–C) P-Mad (A), Brk (B), and *dad*-GFP (C) profiles averaged per TC in relative positions with 15% ventral offset. Error bars represent the standard error per TC at every relative position. (A′–C′) Cells at a given relative position do not experience an increase in P-Mad levels over time, while Brk levels increase 4–5-fold in most of the field. A 2-fold increase in *dad*-GFP levels is only seen in the medial 25% of the disc. Given a relative position x/L_p_, the log-concentration as a function of L_p_ was plotted for each disc (not shown). The linear regression yields an estimate of dlog(c)/dL_p_, where c is the protein concentration. Here, the relative increase in protein concentration for each of these relative positions in the pouch is shown (Δlog(c) = 1 represents a 100% increase from TC1 to TC5): 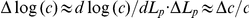, where 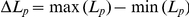.(PDF)

## Abbreviations

AEL: after egg laying
A/P: anterior-posterior
Brk: Brinker
Dad: Daughters against Dpp
Dpp: Decapentaplegic
D/V: dorso-ventral
Mad: Mothers against Dpp
Omb: Optomoter blind
P-Mad: phospho-Mad
Ptc: Patched
Sal: Spalt
Shn: Schnurri
TC: time class
Wg: Wingless
