# Supplementary figures and images for: Dpp Signaling Activity Requires Pentagone to Scale with Tissue Size in the Growing Drosophila Wing Imaginal Disc
Source: PLoS Biol. 2011 Oct 25;9(10):e1001182. doi: 10.1371/journal.pbio.1001182 (PMC3201923; doi:10.1371/journal.pbio.1001182)

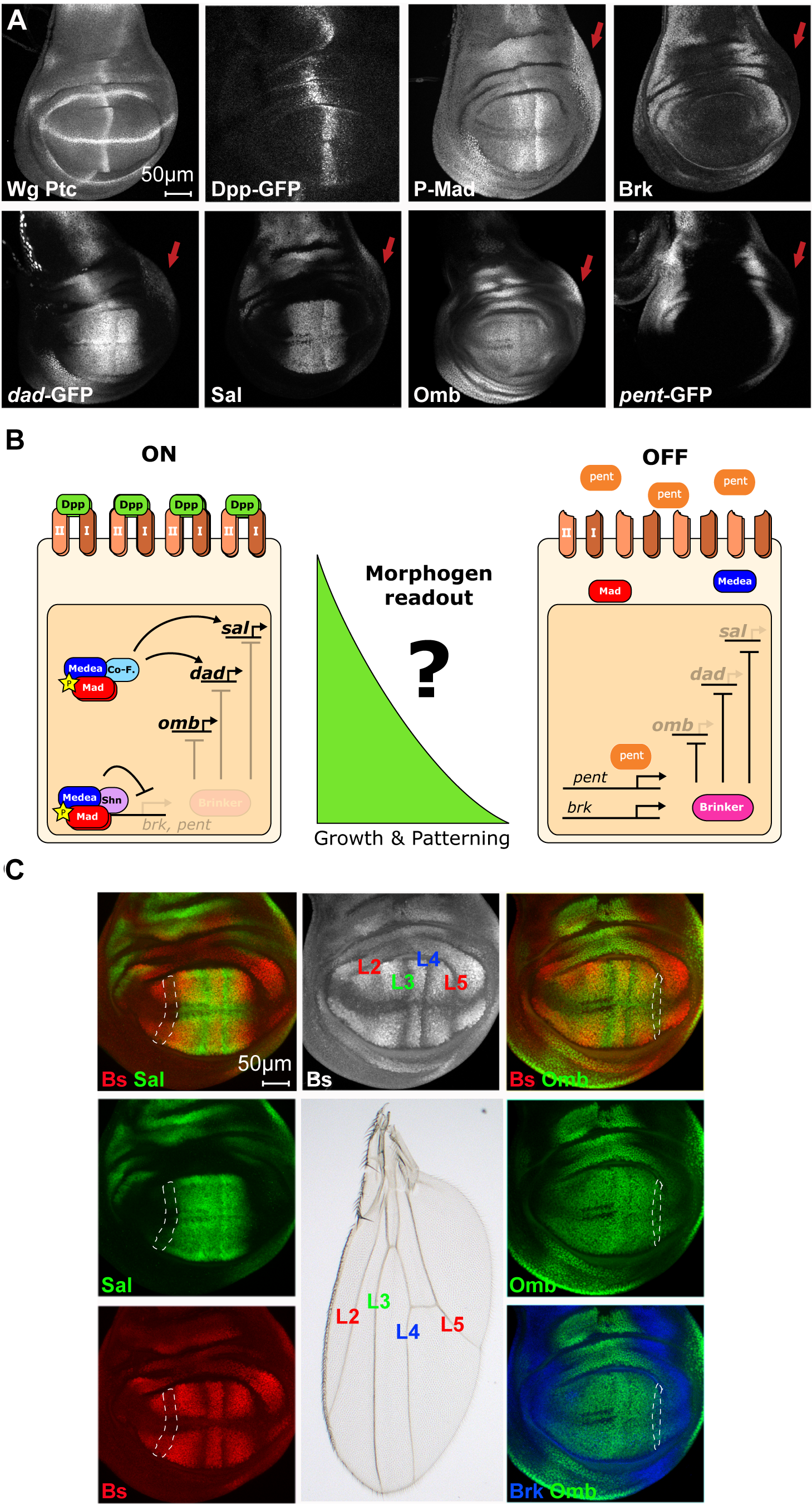

Supplement: Figure S1 — Dpp signal transduction and vein formation. (A) Representative images of expression patterns referred to in this study. Red arrows point to the effect of posterior Dpp source on various target gene expression patterns. (B) In the medial cells (left), type I (Thickveins-Tkv) and type II (Punt) receptors—both Ser/Thr kinases—form heterodimers upon Dpp binding allowing constitutively active Punt to phosphorylate and activate Tkv, which in turn phosphorylates the Mad proteins (Receptor-Smad). P-Mad molecules form complexes with Medea (co-Smad) and translocate into the nucleus where they can both activate as well as inhibit transcription of target genes with the help of co-factors. P-Mad/Medea/Schnurri complex represses transcription of brk and pent via binding to the so-called silencer elements (SEs); hence brk and pent can only be expressed in the lateral cells. Transcription of sal and dad are positively regulated by the P-Mad/Medea complexes. Brk is the default repressor of Dpp target genes, and its removal results in derepression of omb transcription. In the absence of Dpp signal (right), Brk and Pent are expressed at high levels, and Brk keeps sal, dad, and omb off. Pent is secreted and helps movement of Dpp laterally via binding to the HSPG Dally. In between these two extremes, cells read both P-Mad and Brk gradients, and the sensitivity of enhancers to these two factors as well as others determine their response. Modified from [14], figure template courtesy of Alex Weiss. (C) Third instar wing imaginal discs stained for Blistered (Bs) (red) and Sal (green) on the left and for Bs (red), Omb (green), and Brk (blue) on the right. Bs expression is suppressed in the future veins. The middle panel shows the vein positions in a third instar disc and an adult wing. L2, marked with white tracing on the left, is formed within the anterior edge of the Sal/Salr expression domain, overlapping with very low Sal/Salr levels [28]. The L5 primordium, marked with white tracing on [file pbio.1001182.s001.tif]

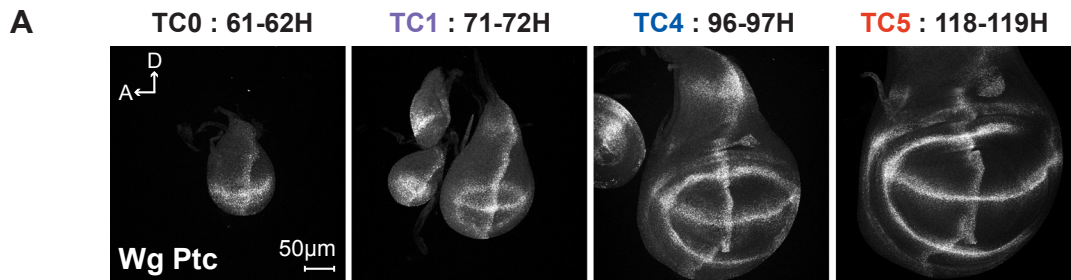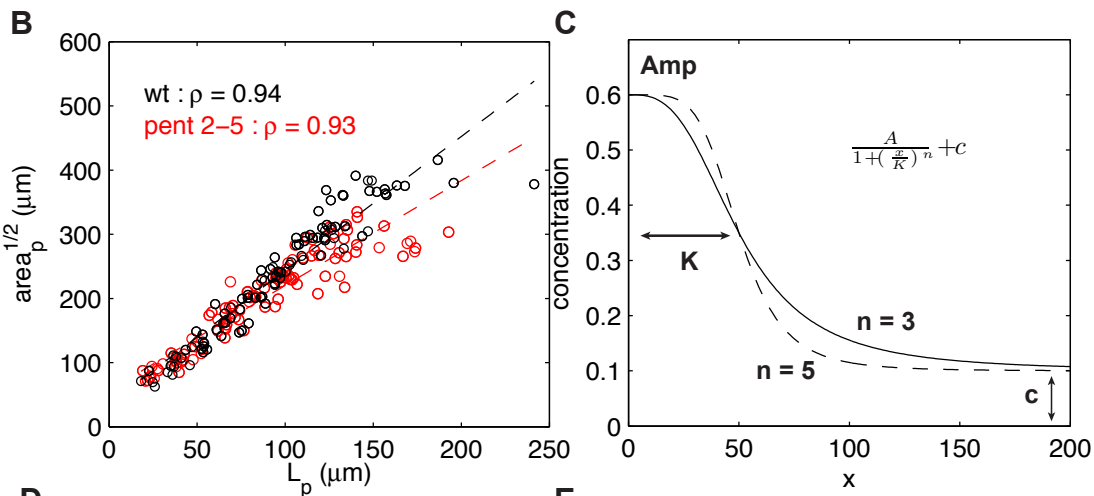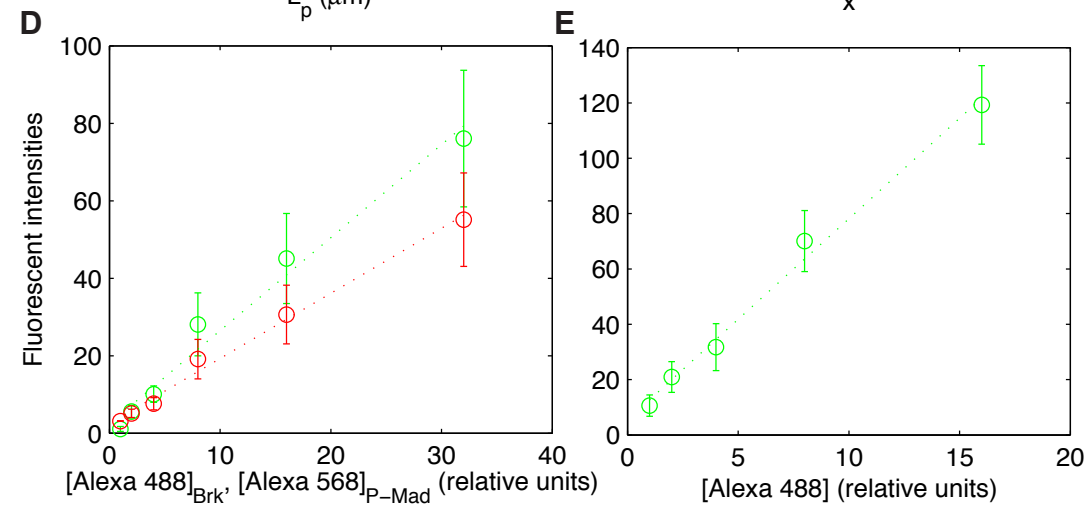

Supplement: Figure S2 — Methods. (A) Discs of varying ages stained with Wg and Ptc antibodies. Wg staining gets refined by 71–72 h AEL. (B) The posterior compartment length measured along the D/V axis (Lp) correlates well with the square root of the posterior compartment area (areap) both in wt (black) and pent2–5 mutant discs (red). Each dot represents a disc. (C) The Hill function used to fit the gene expression domains returns four parameters: the amplitude Amp, the spread of the domain K, the sharpness of the domain boundary n, and a constant offset c. (D–E) Linear range imaging for P-Mad/Brk dataset 1 (D) and Omb/Brk dataset 2 (E). Several dilutions of the secondary antibodies Alexa 488 (green) and Alexa 568 (red) yield fluorescent intensities that are proportional to their concentrations under our imaging conditions. Mean intensities in the whole field and the standard deviations were obtained using the Histogram function in ImageJ. We measured background by imaging an empty slide and subtracted this value. Linear regressions are indicated with dotted lines. (PDF) [file pbio.1001182.s002.pdf]

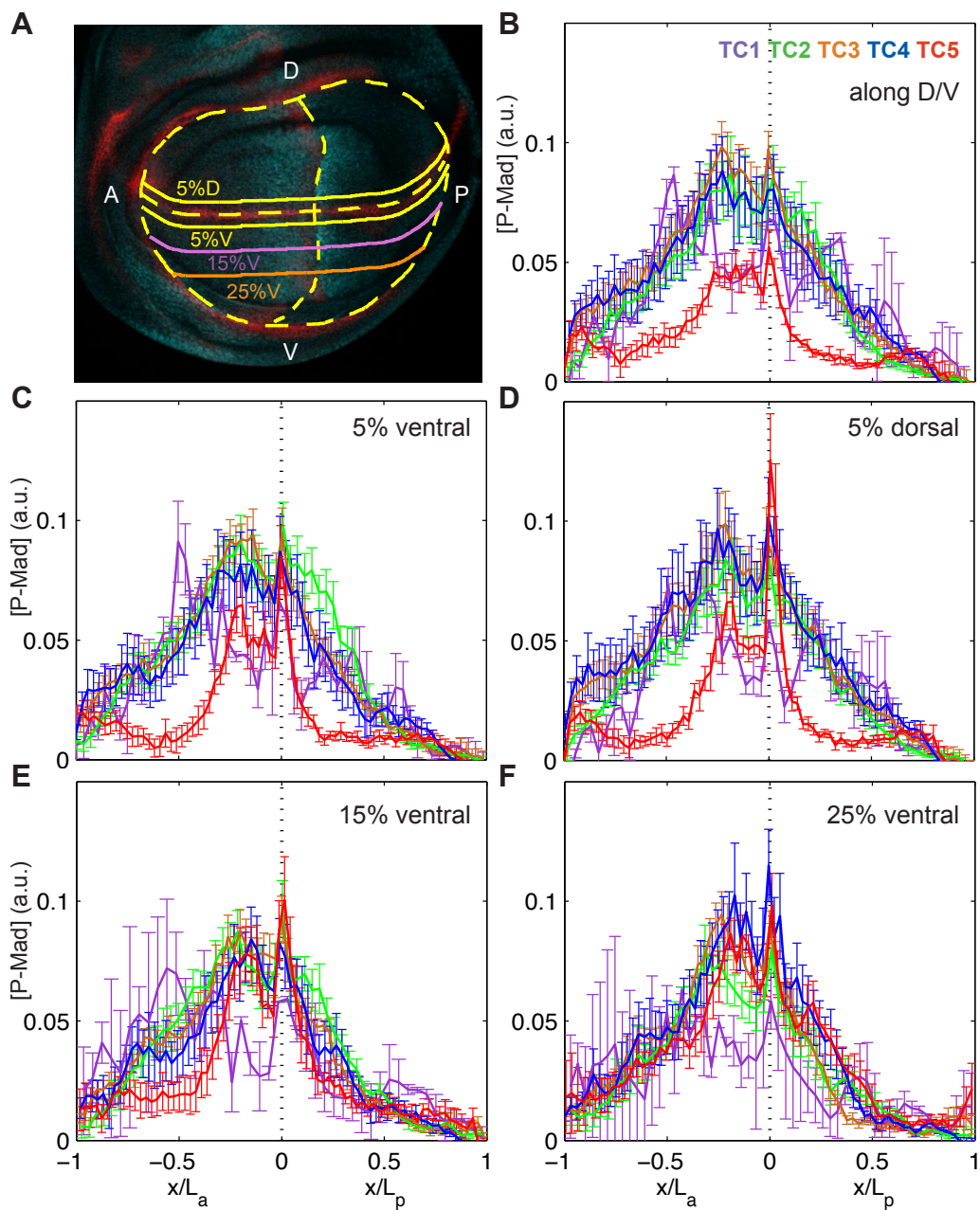

Supplement: Figure S3 — P-Mad is repressed along the D/V boundary at the end of third instar. (A) The dashed yellow lines outline the pouch as well as the A/P and the D/V compartment boundaries, as defined by Wg and Ptc stainings in red. P-Mad profiles were extracted along the D/V and with 5% (yellow), 15% (purple), 25% (orange) offsets from it. (B–F) P-Mad profiles averaged per TC in relative positions along the D/V (B), with 5% offset into the dorsal compartment (D), and with 5% (C), 15% (E), and 25% (F) offsets into the ventral compartment. Positions in the posterior compartment are normalized relative to the posterior compartment length Lp, while positions in the anterior compartment are normalized relative to the anterior compartment length La. (PDF) [file pbio.1001182.s003.pdf]

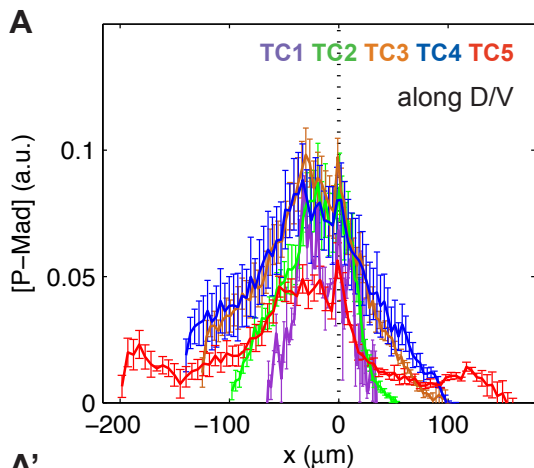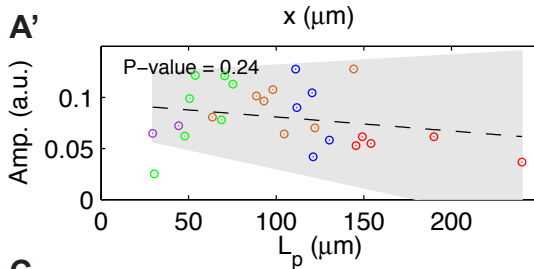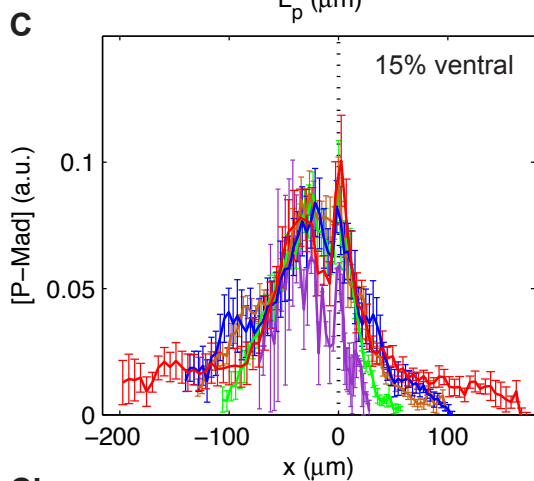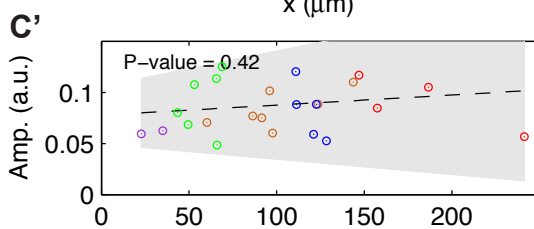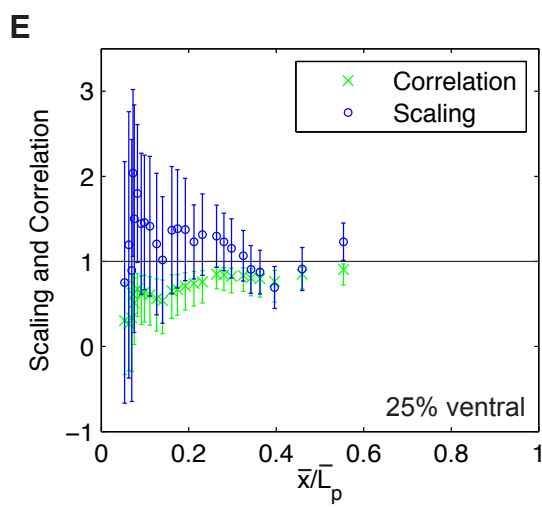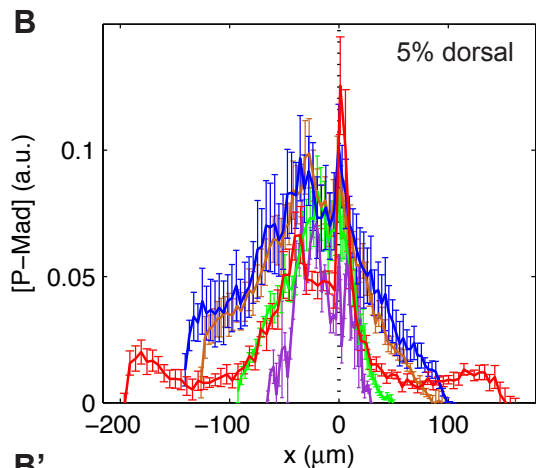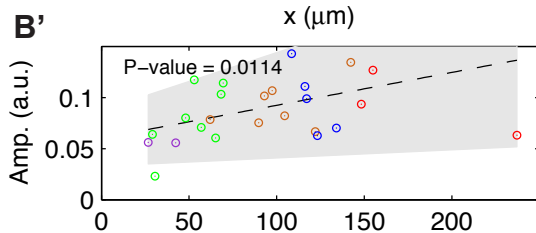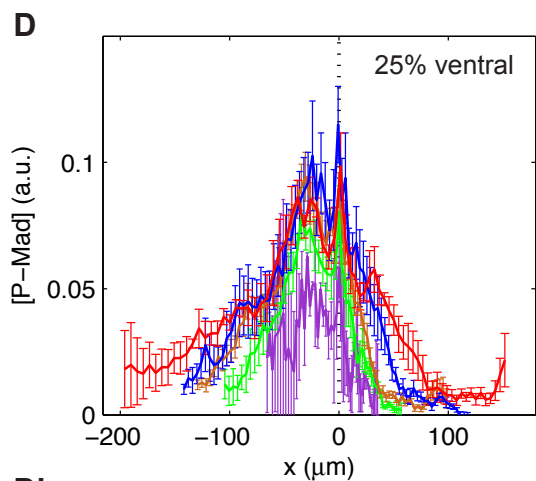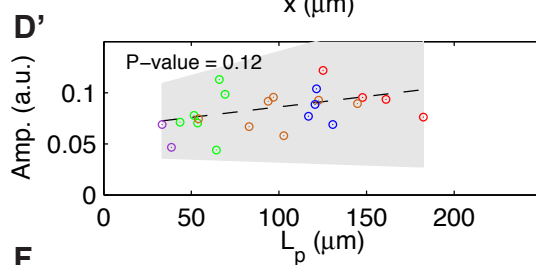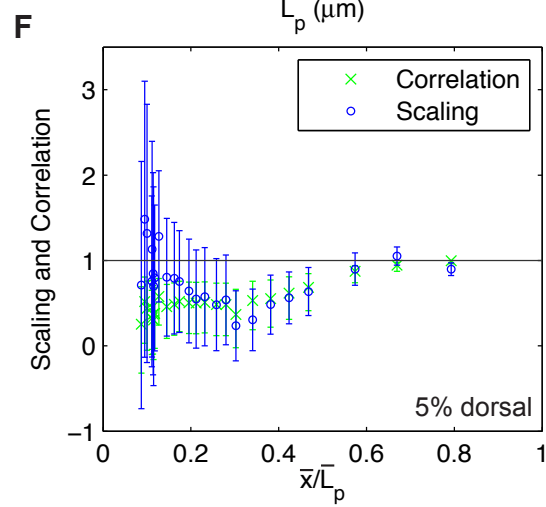

Supplement: Figure S4 — P-Mad profiles and amplitudes at various positions. (A–D) P-Mad profiles averaged per TC along the D/V (A), and with 5% dorsal (B), 15% ventral (C), 25% ventral (D) offsets. (A′–D′) The amplitude of the P-Mad profile (i.e. the concentration at A/P compartment boundary, x = 0) plotted versus the posterior compartment length. Each dot represents a disc and is color-coded according to its age. The linear regression with 95% confidence interval (gray area) and its t test p value under the null hypothesis that the slope is equal to zero are shown. (E–F) P-Mad scaling (o) and correlation (x) for several threshold concentrations using the P-Mad profiles that were extracted with 25% ventral offset (E) and 5% dorsal offset (F). Error bars represent the 95% confidence intervals, obtained from the linear regressions in the case of scaling. (PDF) [file pbio.1001182.s004.pdf]

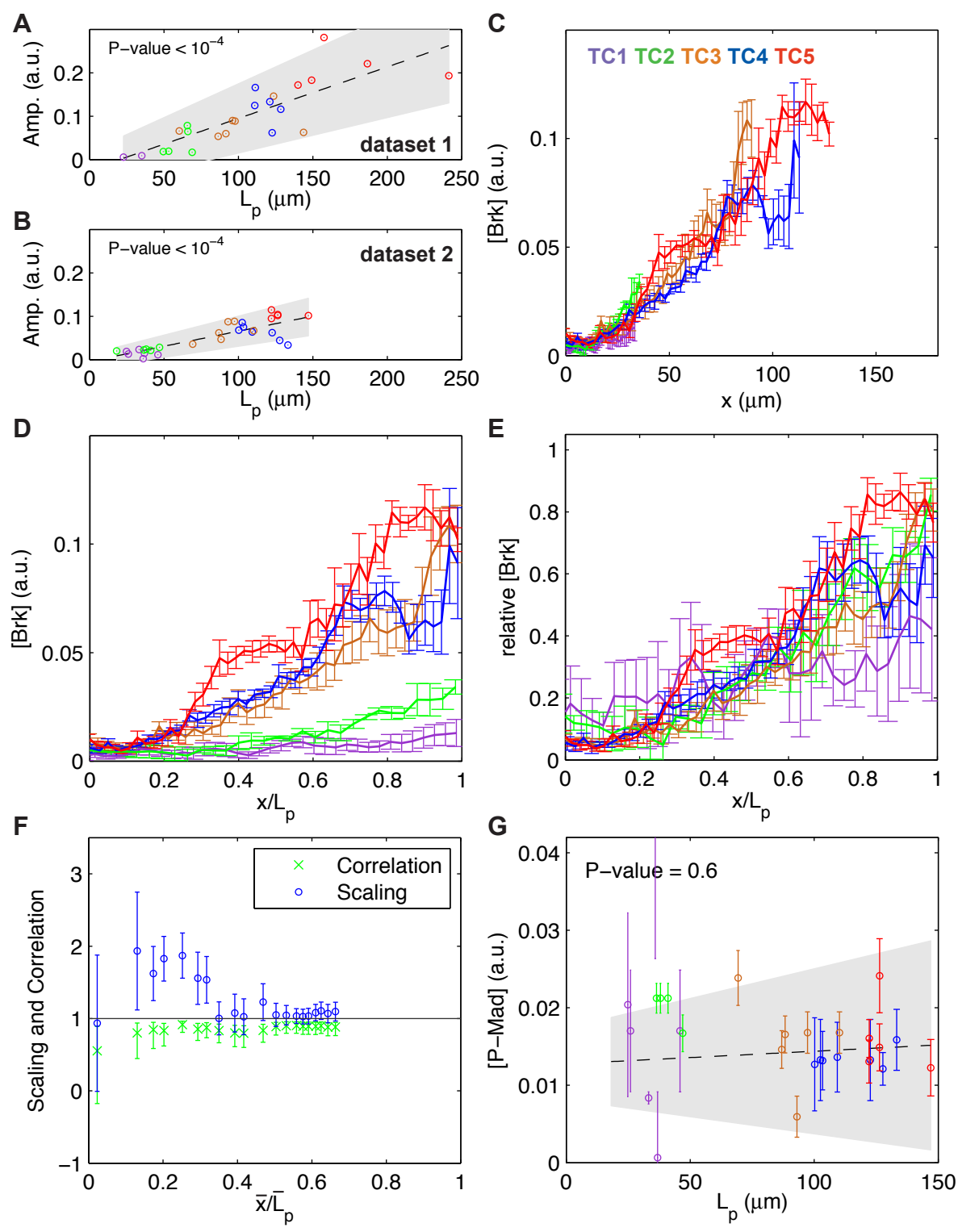

Supplement: Figure S5 — A second dataset for Brk. (A–B) The amplitudes of the Brk profiles (i.e. the peak concentration in the lateral region) at 15% ventral offset versus the posterior compartment length for dataset 1 (A) and dataset 2 (B). Taking the extremes, the ratio between the extreme values of the Brk amplitudes (Amp) are max(Amp)/min(Amp) = 48.7 for dataset 1 and max(Amp)/min(Amp) = 48.3 for dataset 2, respectively. (C) Brk profiles averaged per TC with 15% ventral offset (dataset 2). (D) Profiles in (C) in relative positions. (E) Profiles in (D) with normalized amplitudes. (F) Brk scaling for several threshold concentrations. Error bars represent the 95% confidence intervals. To compute scaling at each position, the Brk profiles with normalized amplitudes were used (dataset 2). (G) Brk profiles were fitted with a decaying exponential function (dataset 2) to obtain the decay length λBrk of the profile. For each disc, the average P-Mad concentration at the position x = λBrk was plotted against the Lp of the disc. The weighted linear regression with 95% confidence interval (gray area) and its t test p value under the null hypothesis that the slope is equal to zero are shown. (PDF) [file pbio.1001182.s005.pdf]

**A**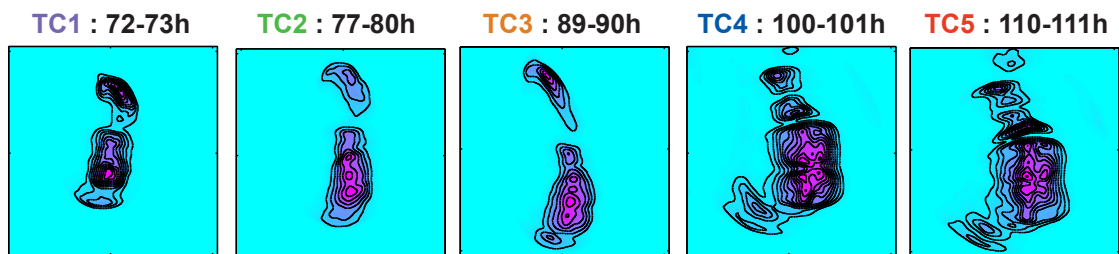**B**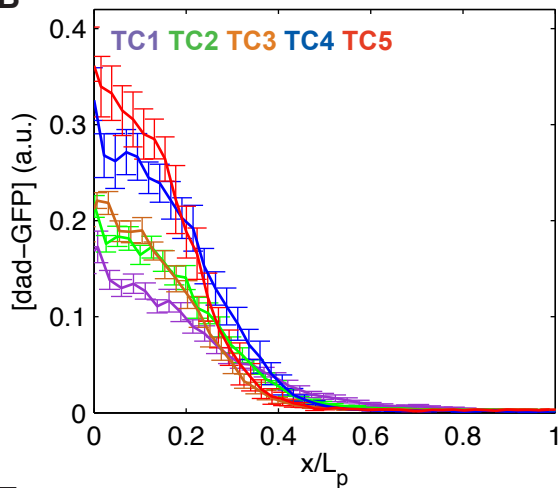**C**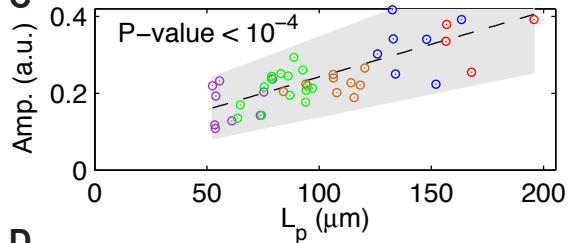**D**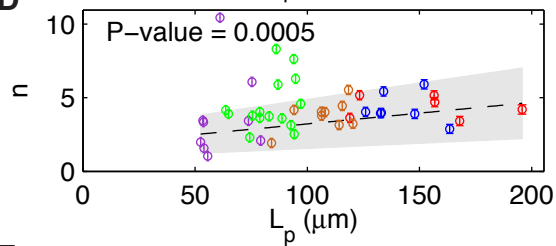**E**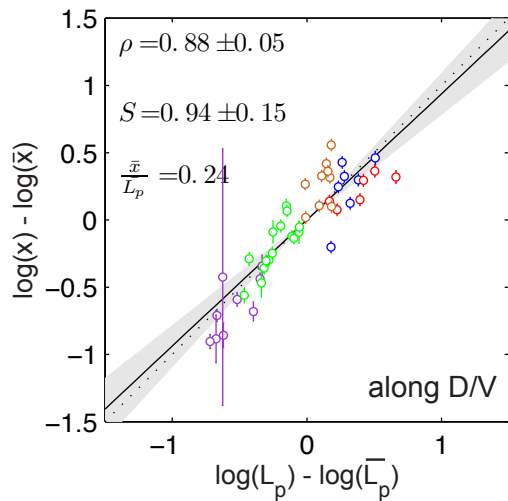**F**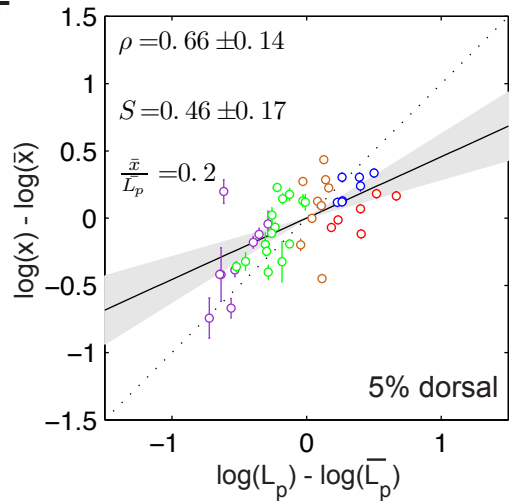**G**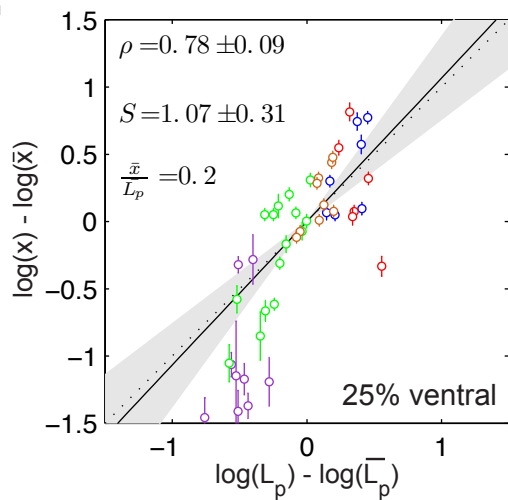**H**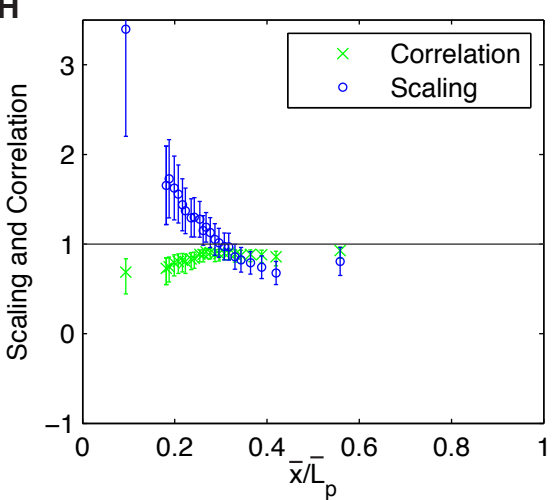

Supplement: Figure S6 — dad-GFP shows position-dependent scaling. (A) For each time class, a representative dad-GFP contour plot is shown. Lower concentrations are in light blue, higher concentrations in pink. (B) dad-GFP profiles averaged per TC in relative positions, at 15% ventral offset. (C) The amplitudes of the dad-GFP profiles (i.e. the concentration at A/P compartment boundary, x = 0) plotted against the Lp for each disc, at 15% ventral offset. (D) dad-GFP domain boundary at 15% ventral offset gets slightly sharper across growth. Error bars represent 95% confidence intervals from the Hill fits (see also Figure S2C). The weighted linear regression with 95% confidence interval (gray area) and its t test p value under the null hypothesis that the slope is equal to zero are shown. (E–G) Scaling and correlation of dad-GFP domain boundary along the D/V (E), and with 5% dorsal (F), 25% ventral (G) offsets. (H) Scaling of dad-GFP for several threshold concentrations when treated as a gradient. Error bars represent the 95% confidence intervals, obtained from the linear regressions in the case of scaling. Consistent with the profiles in (B), positions anterior to ∼0.3 Lp show hyper-scaling due to the increasing dad-GFP levels, while positions posterior to it show hypo-scaling. (PDF) [file pbio.1001182.s006.pdf]

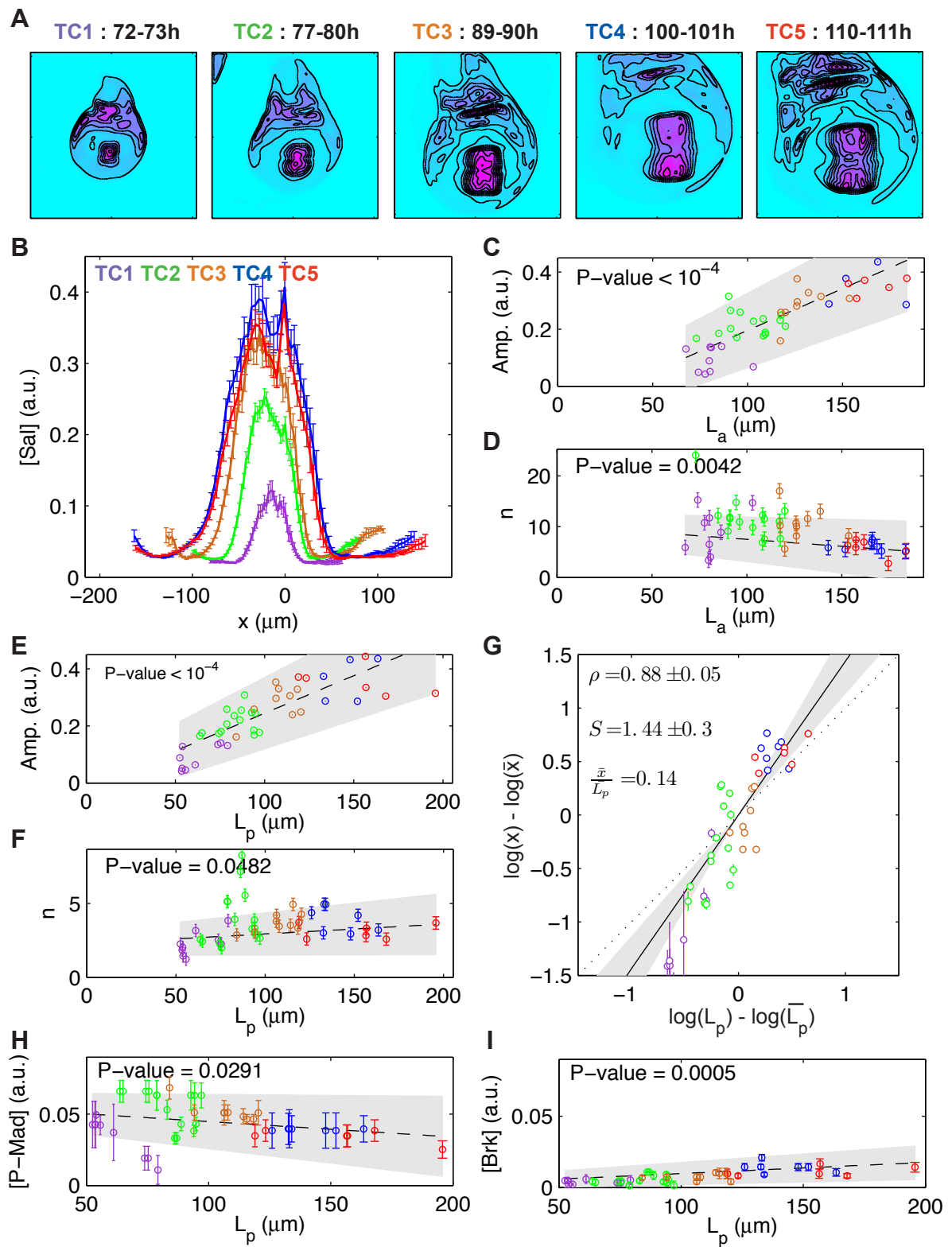

Supplement: Figure S7 — Sal amplitudes increase over time and the posterior Sal domain hyper-scales. (A) Representative Sal contour plots for each TC. Lower concentrations are in light blue, higher concentrations in pink. (B) Sal profiles averaged per TC at 15% ventral offset. (C, E) Sal amplitudes (i.e. the maximum concentration in the vicinity of the A/P compartment boundary at x = 0) for each disc at 15% ventral offset versus the anterior (C) and posterior (E) compartment lengths. (D, F) Sharpness of the Sal domain boundary (n) in the anterior (D) and the posterior (F) compartments plotted against tissue size. (G) Scaling and correlation of the Sal domain boundary in the posterior compartment at 15% ventral offset. (H–I) The average P-Mad (H) and Brk (I) concentrations at the position x = KSal_P (Sal boundary in the posterior compartment) were plotted against the Lp for each disc. The weighted linear regression with 95% confidence interval (gray area) and its t test p value under the null hypothesis that the slope is equal to zero are also shown. (PDF) [file pbio.1001182.s007.pdf]

**A**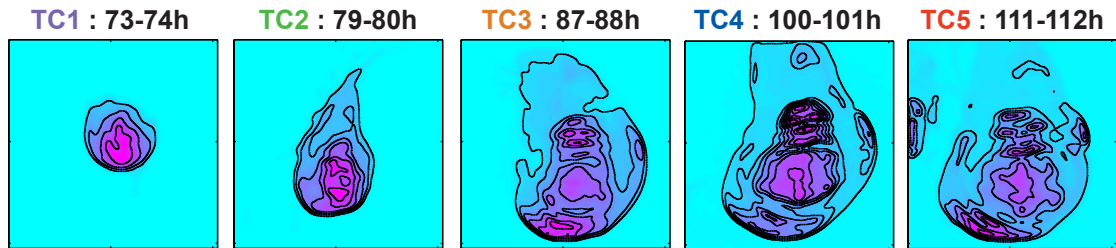**B**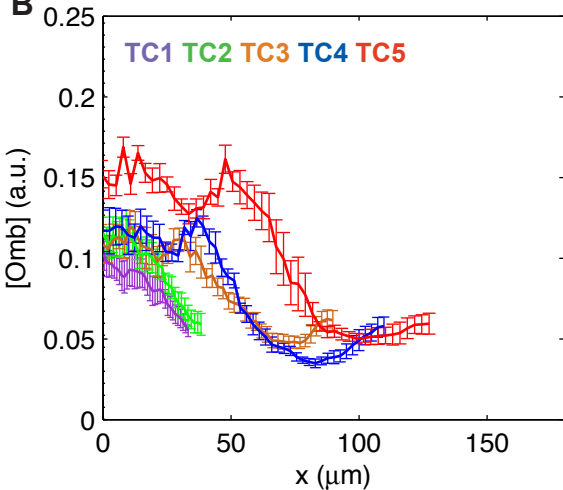**C**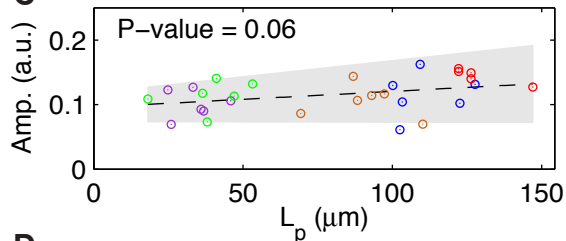**D**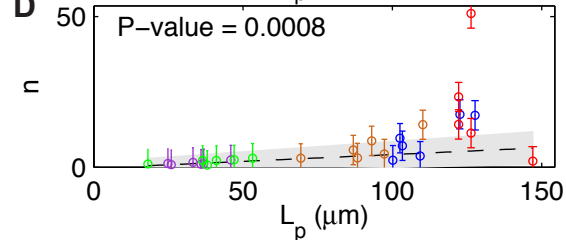

Supplement: Figure S8 — Omb domain scales with tissue size. (A) Representative Omb contour plots for each TC. Lower concentrations are in light blue, higher concentrations in pink. (B) Omb profiles averaged per TC at 15% ventral offset. (C) Omb amplitudes (i.e. the concentration at A/P compartment boundary, x = 0) versus the posterior compartment length for each disc at 15% ventral offset. The linear regression with 95% confidence interval (gray area) and its t test p value under the null hypothesis that the slope is equal to zero are also shown. (D) Sharpness of the Omb domain boundary. The weighted linear regression with 95% confidence interval (gray area) and its t test p value under the null hypothesis that the slope is equal to zero are also shown. (PDF) [file pbio.1001182.s008.pdf]

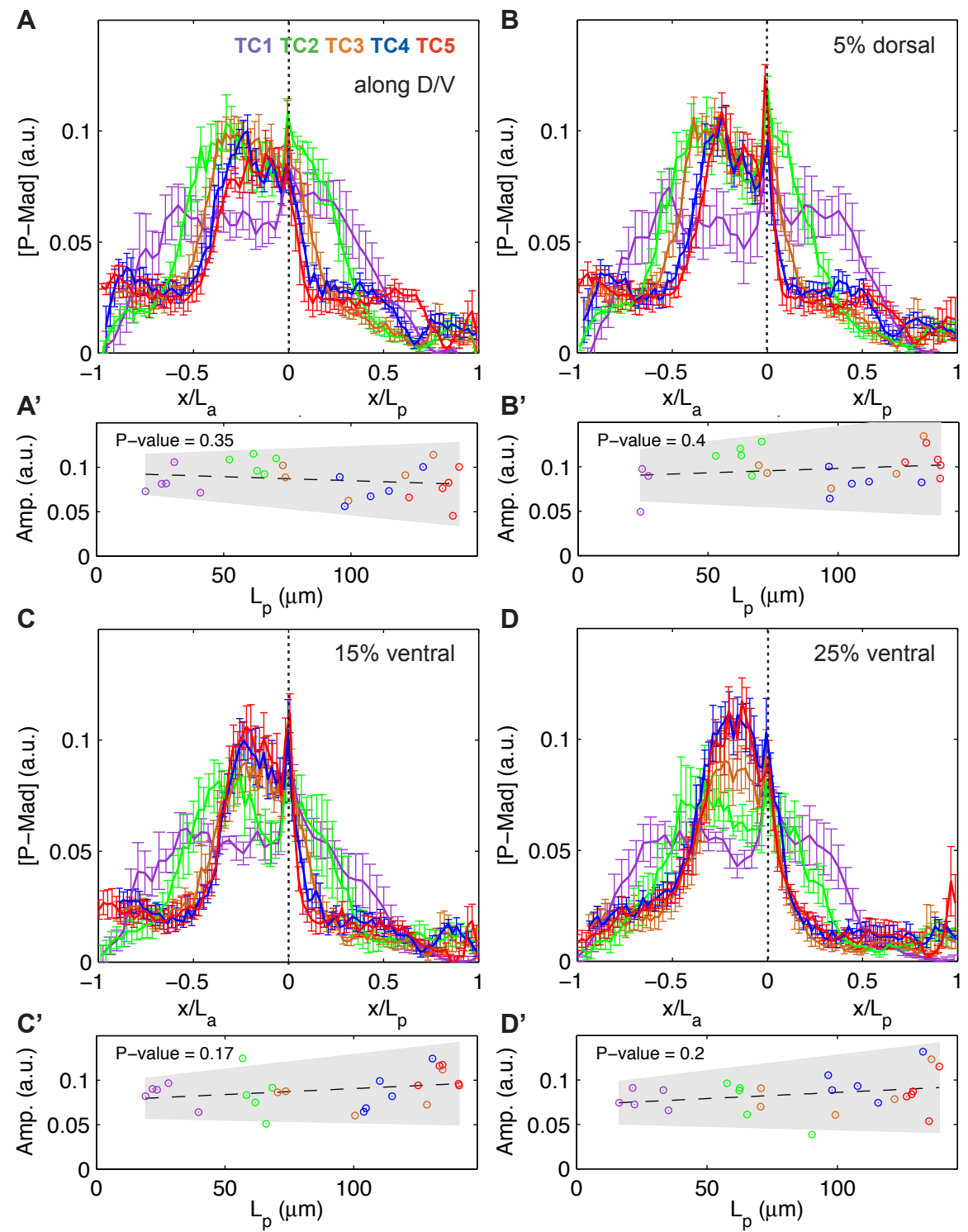

Supplement: Figure S9 — Lack of size adaptation of P-Mad profiles in pent2–5. (A–D) P-Mad profiles in pent2–5 mutant background are shown in relative positions and averaged per TC along the D/V (A), and with 5% dorsal (B), 15% ventral (C), 25% ventral (D) offsets. Positions in the posterior compartment are normalized relative to the posterior compartment length Lp, while positions in the anterior compartment are normalized relative to the anterior compartment length La. (A′–D′) The amplitude of the P-Mad profile in pent2–5 mutant background (i.e. the concentration at A/P compartment boundary, x = 0) was plotted versus the posterior compartment length for each disc. The linear regression with 95% confidence interval (gray area) and its t test p value under the null hypothesis that the slope is equal to zero are also shown. (PDF) [file pbio.1001182.s009.pdf]

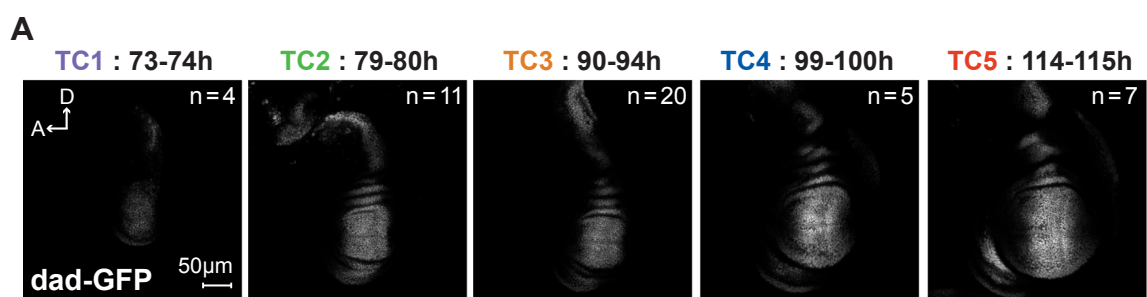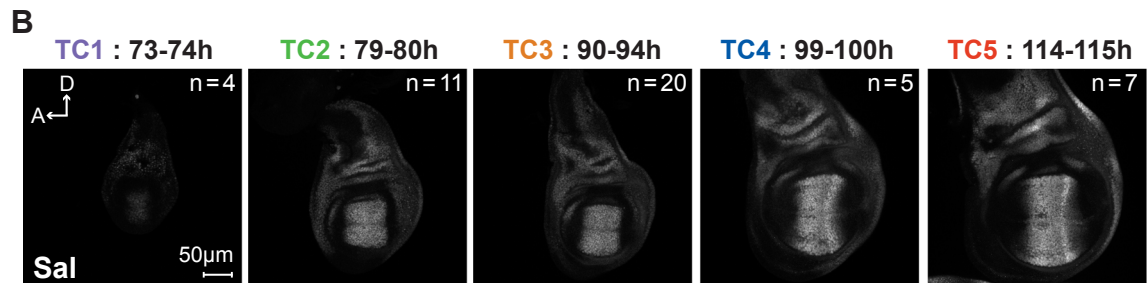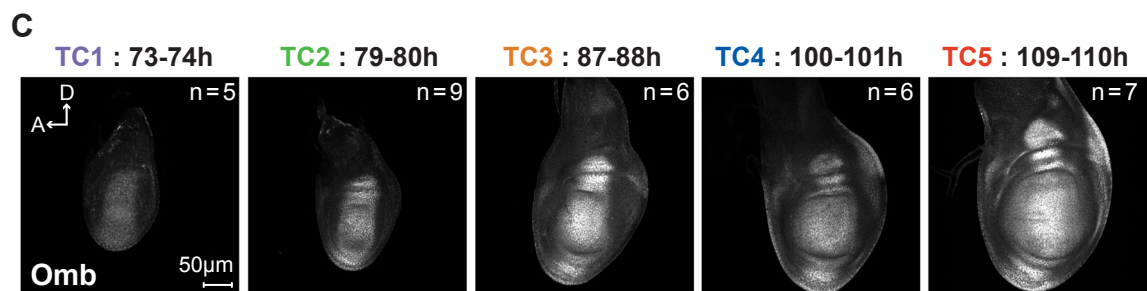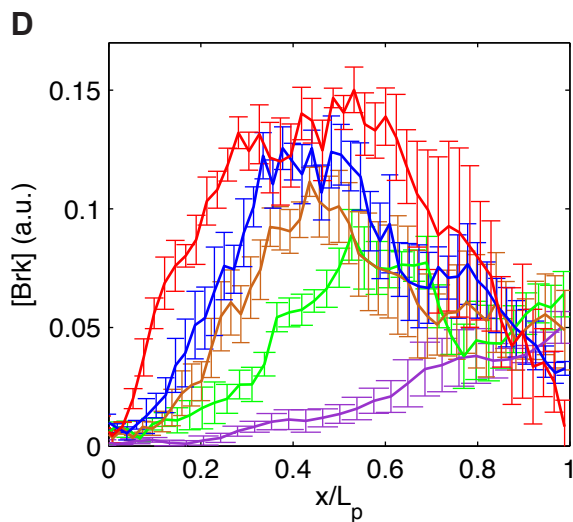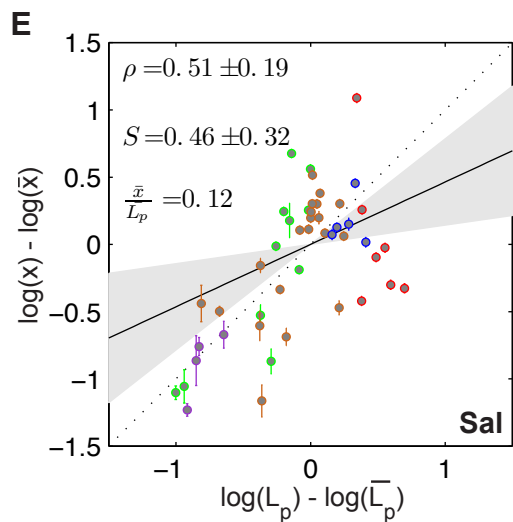

Supplement: Figure S10 — Expression patterns of target genes in pent2–5. (A–C) Representative images of dad-GFP (A), Sal (B), and Omb (C) in pent2–5 mutant background. (D) Brk profiles averaged per TC in relative positions at 15% ventral offset in pent2–5 background. Brk levels still increase in pent mutants and the profiles move relatively inwards as the discs grow. (E) Posterior Sal domain boundary does not scale in pent2–5 discs. (PDF) [file pbio.1001182.s010.pdf]

*wild-type*

*pent2-5*

*brkXA+/-;pent2-5*

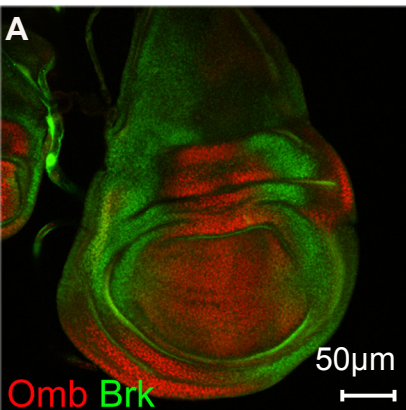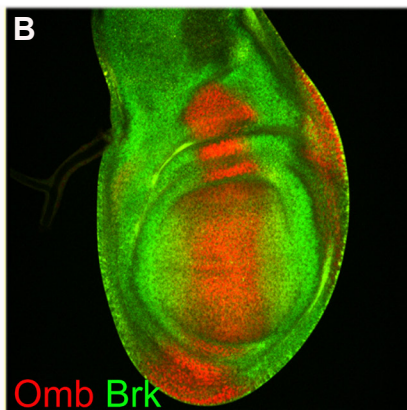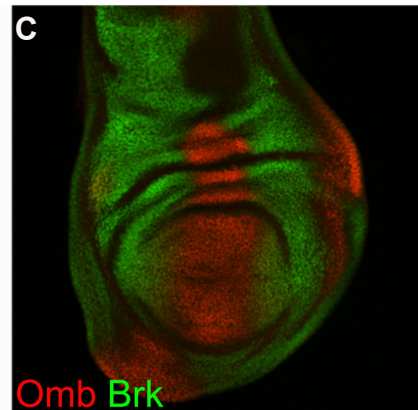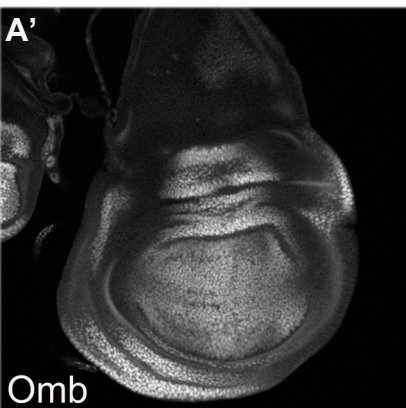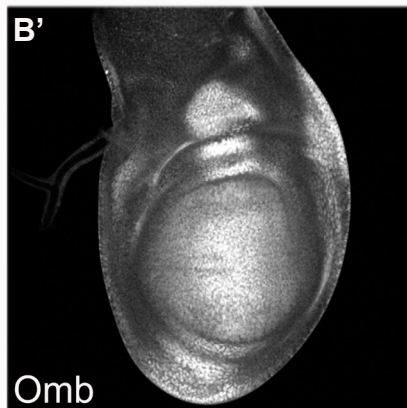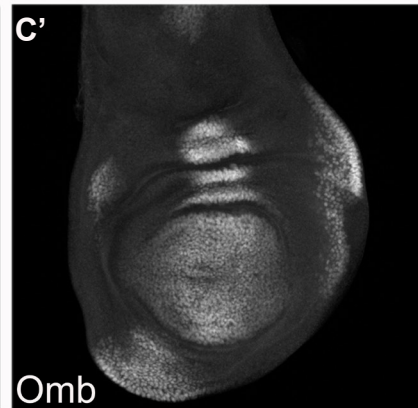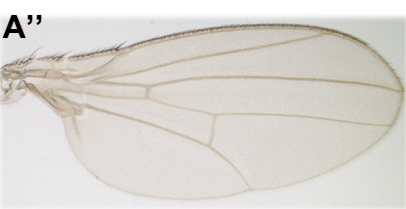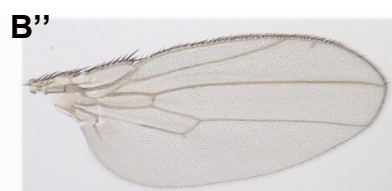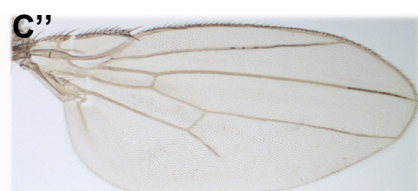

Supplement: Figure S11 — Omb and Brk domains overlap extensively in pent2–5, and heterozygosity for brk rescues growth defects of pent mutants. (A–A″) wt (B–B″) pent2–5 (C–C″) brkXA+/−; pent2–5. (A, B, C) Third instar wing imaginal discs stained for Omb (red) and Brk (green). (A′, B′, C′) Omb channel only (gray). (A″, B″, C″) Representative wings from female flies of corresponding genotypes. Heterozygosity for brk rescues growth defects of pent mutants to a large extent. The slight rescue of L5 shown in C″ is highly variable and represents an average wing; some flies have an almost complete rescue of L5 while others display no rescue. (PDF) [file pbio.1001182.s011.pdf]

**A**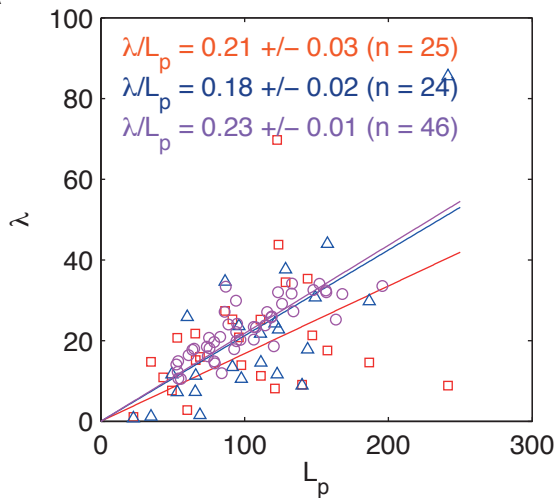**B**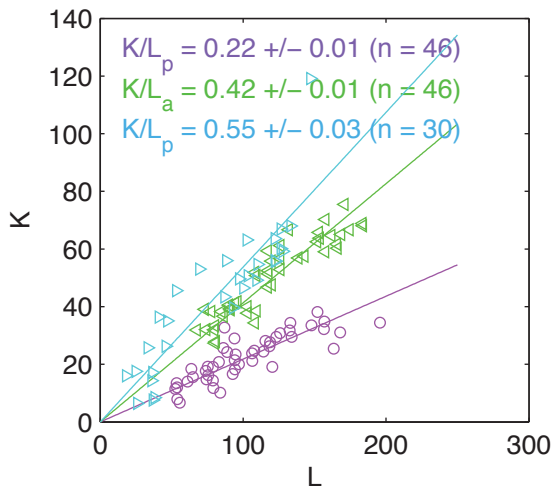

Supplement: Figure S12 — Decay length λ correlates with tissue size. (A) P-Mad, Brk, and dad-GFP profiles were fitted with an exponential and the resulting decay lengths (λ) were plotted as a function of the posterior compartment length Lp. The text in the plot shows the average λ/Lp ratio with its standard error. Relationships obtained from the linear regression are displayed in the boxes on the right. Note that Lp is measured along the D/V compartment boundary. (B) Same as (A), but the dad-GFP, Sal, and Omb profiles were fitted with a Hill function instead of an exponential. (PDF) [file pbio.1001182.s012.pdf]

**A**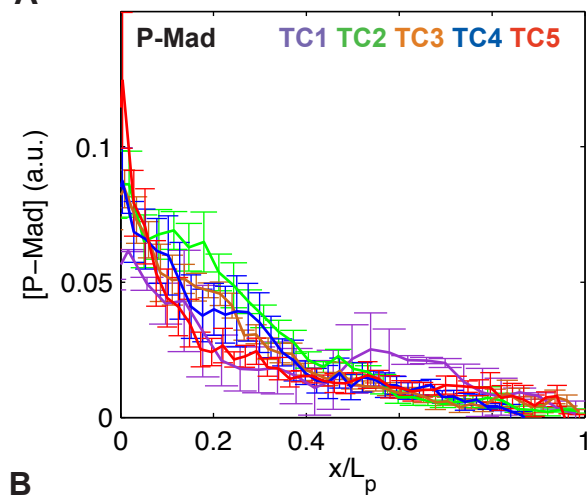**B**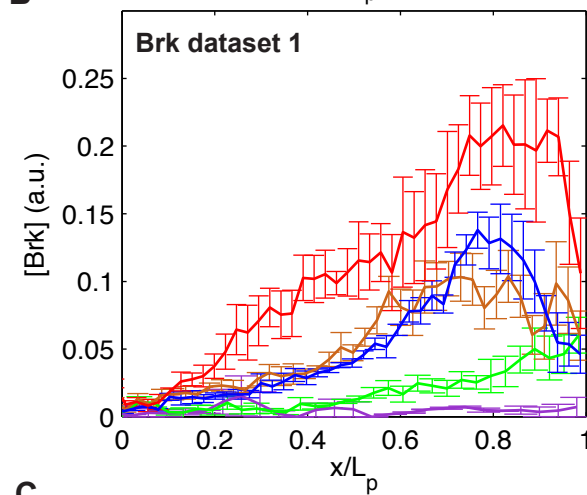**C**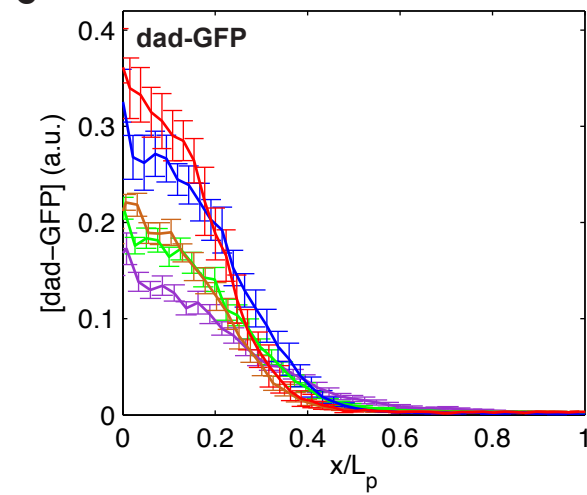**A'**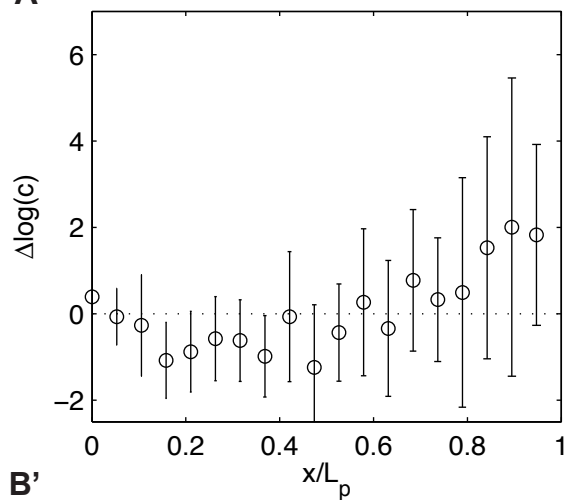**B'**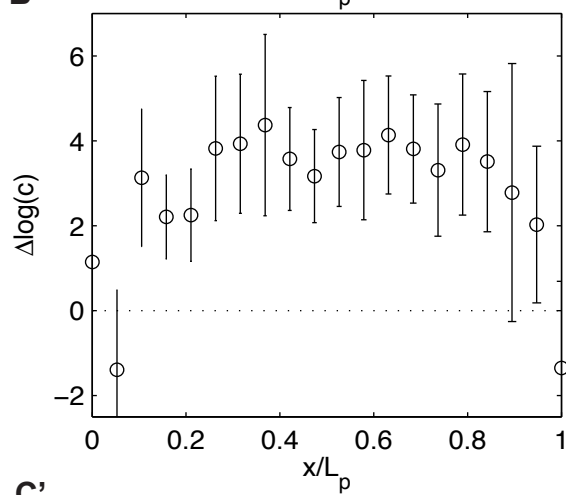**C'**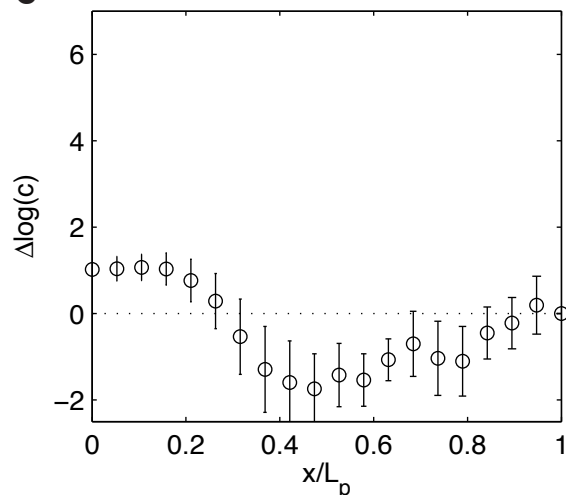

Supplement: Figure S13 — Changes in P-Mad, Brk, and dad-GFP levels at relative positions. (A–C) P-Mad (A), Brk (B), and dad-GFP (C) profiles averaged per TC in relative positions with 15% ventral offset. Error bars represent the standard error per TC at every relative position. (A′–C′) Cells at a given relative position do not experience an increase in P-Mad levels over time, while Brk levels increase 4–5-fold in most of the field. A 2-fold increase in dad-GFP levels is only seen in the medial 25% of the disc. Given a relative position x/Lp, the log-concentration as a function of Lp was plotted for each disc (not shown). The linear regression yields an estimate of dlog(c)/dLp, where c is the protein concentration. Here, the relative increase in protein concentration for each of these relative positions in the pouch is shown (Δlog(c) = 1 represents a 100% increase from TC1 to TC5): , where . (PDF) [file pbio.1001182.s013.pdf]
